# Comparative genome analysis of non-toxigenic non-O1 versus toxigenic O1 *Vibrio cholerae*

**DOI:** 10.7243/2052-7993-2-1

**Published:** 2014

**Authors:** Munmun Mukherjee, Prathusha Kakarla, Sanath Kumar, Esmeralda Gonzalez, Jared T. Floyd, Madhuri Inupakutika, Amith Reddy Devireddy, Selena R. Tirrell, Merissa Bruns, Guixin He, Ingrid E. Lindquist, Anitha Sundararajan, Faye D. Schilkey, Joann Mudge, Manuel F. Varela

**Affiliations:** 1 Eastern New Mexico University, Department of Biology, Portales, New Mexico, 88130, USA.; 2 QC Laboratory, Harvest and Post Harvest Technology Division, Central Institute of Fisheries Education (CIFE), Seven Bungalows, Versova, Andheri (W), Mumbai 400061, India.; 3 University of Massachusetts Lowell, Department of Clinical Laboratory and Nutritional Sciences, Lowell, MA 01854, USA.; 4 National Center for Genome Resources, Santa Fe, New Mexico, 87505, USA.

**Keywords:** *Vibrio cholerae*, O1, non-O1, serogroup, cholera, cholera toxin, virulence, genome comparison

## Abstract

Pathogenic strains of *Vibrio cholerae* are responsible for endemic and pandemic outbreaks of the disease cholera. The complete toxigenic mechanisms underlying virulence in *Vibrio* strains are poorly understood. The hypothesis of this work was that virulent versus non-virulent strains of *V. cholerae* harbor distinctive genomic elements that encode virulence. The purpose of this study was to elucidate genomic differences between the O1 serotypes and non-O1 *V. cholerae* PS15, a non-toxigenic strain, in order to identify novel genes potentially responsible for virulence. In this study, we compared the whole genome of the non-O1 PS15 strain to the whole genomes of toxigenic serotypes at the phylogenetic level, and found that the PS15 genome was distantly related to those of toxigenic *V. cholerae*. Thus we focused on a detailed gene comparison between PS15 and the distantly related O1 *V. cholerae* N16961. Based on sequence alignment we tentatively assigned chromosome numbers 1 and 2 to elements within the genome of non-O1 *V. cholerae* PS15. Further, we found that PS15 and O1 *V. cholerae* N16961 shared 98% identity and 766 genes, but of the genes present in N16961 that were missing in the non-O1 *V. cholerae* PS15 genome, 56 were predicted to encode not only for virulence–related genes (colonization, antimicrobial resistance, and regulation of persister cells) but also genes involved in the metabolic biosynthesis of lipids, nucleosides and sulfur compounds. Additionally, we found 113 genes unique to PS15 that were predicted to encode other properties related to virulence, disease, defense, membrane transport, and DNA metabolism. Here, we identified distinctive and novel genomic elements between O1 and non-O1 *V. cholerae* genomes as potential virulence factors and, thus, targets for future therapeutics. Modulation of such novel targets may eventually enhance eradication efforts of endemic and pandemic disease cholera in afflicted nations.

## Introduction

Cholera is an infectious disease characterized by profuse watery diarrhea and vomiting in humans, and the causative agent is *Vibrio cholerae,* a Gram-negative, comma-shaped, facultative anaerobic bacterium [1]. *V. cholerae* includes both pathogenic and non-pathogenic strains, and the bacteria responsible for pandemic outbreaks secrete the cholera toxin [2]. Since 1817, seven pandemics of cholera have been recorded. Cholera is a major public health concern because the disease can exhibit significant mortality if left untreated [3,4]. In the past 200 years, cholera has resulted in millions of deaths due to its ability to spread rapidly within populations, and has been capable of contaminating rivers and estuaries [5]. The most recent outbreak of *V. cholerae* was recorded in Southeast Asia, which quickly spread across the globe as the seventh pandemic [6]. In 2010 alone, 604,634 cases of cholera were reported in Haiti, raising the death toll count to 7,436 in the first two years [7].

The genomes of several pathogenic *V. cholerae* strains encode proteins that are directly or indirectly responsible for virulence. In many parts of the world, the O serogroups of *V. cholerae* are associated with diarrhea [8]. The most common mode of transmission for this bacterium is through the consumption of feces-contaminated water, fishes or crustaceans [9]. In addition to rehydration therapy, the first line of antimicrobial agent used against cholera is doxycycline, prescribed for a period of 1-3 days in order to reduce the severity of the symptoms [10,11]. Other antimicrobials which have been demonstrated to be effective in humans include cotrimoxazole, erythromycin, tetracycline, chloramphenicol, furazolidone and norfloxacin [11,12].

Unfortunately, wide spread use and misuse of these and other antimicrobials have resulted in selection of multidrug-resistant bacterial variants [13] which potentially compromise chemotherapeutic efficacy towards cholera [14]. The different mechanisms by which bacteria show resistance to antimicrobial agents include (a) biofilm production (b) drug inactivation (c) ribosome protection (d) reduced permeability (e) target alteration [15] and (f) active efflux [16]. One of the active efflux pumps of *V. cholerae* is EmrD-3, which belongs to the major facilitator superfamily (MFS) and is a drug/H^+^ antiporter with 12 transmembrane domains [17]. Another efflux pump encoded in the genome of *V. cholerae* is VceB [18]. Drug efflux pumps are integral membrane transporters that actively efflux the toxic compounds and antibiotics out of the bacterial cell and confer resistance against multiple antibacterial agents [19-21].

The presence of the cholera toxin (CT), the *Vibrio* pathogenicity island (VPI), and the toxin co-regulated pilus (TCP) within the O1 serogroups of *V. cholerae* make these strains more virulent and pandemic than their non-O1 counterparts [22]. A significant basis for their pathogenicity is attributed to cholera toxin encoding genes. Other genes important for enhancing virulence in these organisms are *ace*, *psh*, *PIIICTX*, *zot* and *cep*, which are implicated in phage morphogenesis [5,23,24]. The *Vibrio* pathogenicity island-1 (VPI-1) confers toxin release, bioflim formation, attachment to disease vectors for transmission to humans, and are receptors of CTX. The *Vibrio* pathogenicity island-2 (VPI-2) helps the cholera toxin to gain entry into the intestinal epithelium by unmasking GM1 gangliosides in the lining of the human intestine. The absence of VPI-1 and VPI-2 in non-O1 serogroups of *V. cholerae* makes them less pathogenic than the O1 serogroups [25].

Even though non-O1 *V. cholerae* strains carry certain virulence genes, the severity of disease is less compared to O1/ O139 *V. cholerae* [8]. The non-O1 serogroups of *V. cholerae* are known as the non-agglutinating *Vibrio*s (NAGs) because they lack the genes coding for CT and TCP [26,27]. The presence of multidrug resistance (MDR) transporters confers resistance to ampicillin, chloramphenicol and tetracycline in non-O1 and non-O139 serogroups of *V. cholerae* species [14]. The ABC transporters present in PS15 *V. cholerae* predictably transport phosphate molecules across the periplasm and may be essential for protein synthesis, amino acid exchange, and transport of fatty acids [28].

We previously determined the genome nucleotide sequence of the non-O1 non-toxigenic *V. cholerae* PS15 (GenBank Accession No. AIJR00000000) [28]. Here, we compared non-O1 PS15 with the genetic information of virulent strains. The genome of *V. cholerae* PS15 is composed of 3,910,387 base pairs (bp) organized into 3,512 open reading frames with a G+C content of 47.55% [28]. We chose to focus our comparative analysis with *V. cholerae* PS15 [29] using *V. cholerae* El Tor N16961 because this latter genome was completely sequenced [30]. N16961 is made up of 4,033,460 base pairs (bp) organized and distributed into two chromosomes, with a G+C content of 46.9% in chromosome 1 and 47.7% in chromosome 2 [30]. Even though the non-O1 *V. cholerae* bacterium possesses some virulence genes responsible for causing gastrointestinal infections, wound infections, septicemia and cellulitis in humans, little is known about the mechanisms that confer virulence in this microorganism. The aim of this work is to identify differences in the genetic elements between the genomes of virulent N16961 and non-virulent PS15 strains of *V. cholerae* in order to identify novel virulence mechanisms that may eventually serve as potential therapeutic targets for the ultimate purpose of fostering conditions that reduce dissemination of disease-causing virulent serotypes of *V. cholerae* through populations.

## Methods

### Comparison of non-O1 PS15 and O1 N16961 *Vibrio cholerae* genomes using RAST and UniProt

A function based genome comparison was performed between a non-toxigenic, non-O1 *V. cholerae* PS15 environmental isolate (courtesy of Dr. Charles Kaysner) from sediment sampled in Puget Sound, WA [28,31] and O1 *V. cholerae* N16961 [30], using the RAST (Rapid Annotation using Subsystem Technology) database and Seed Viewer to predict protein function [32] focusing on comparison of categories and subsystem groupings pertaining to virulence, disease, defense, membrane transport, DNA metabolism, regulons, dormancy, sporulation, phages, prophages, transposable elements, and plasmids for both genomes of O1 and non-O1 *V. cholerae* microorganisms. The open reading frames (genes) encoding functional roles associated with a subsystem are referred as functioning parts, and a subsystem is referred as a set of predicted abstract functional roles [32]. The screening of predicted proteins encoded from elements of both genomes was performed with BLAST analysis of the amino acid sequences using UniProt [33].

### Phylogenetic analysis

**Figure 1:**
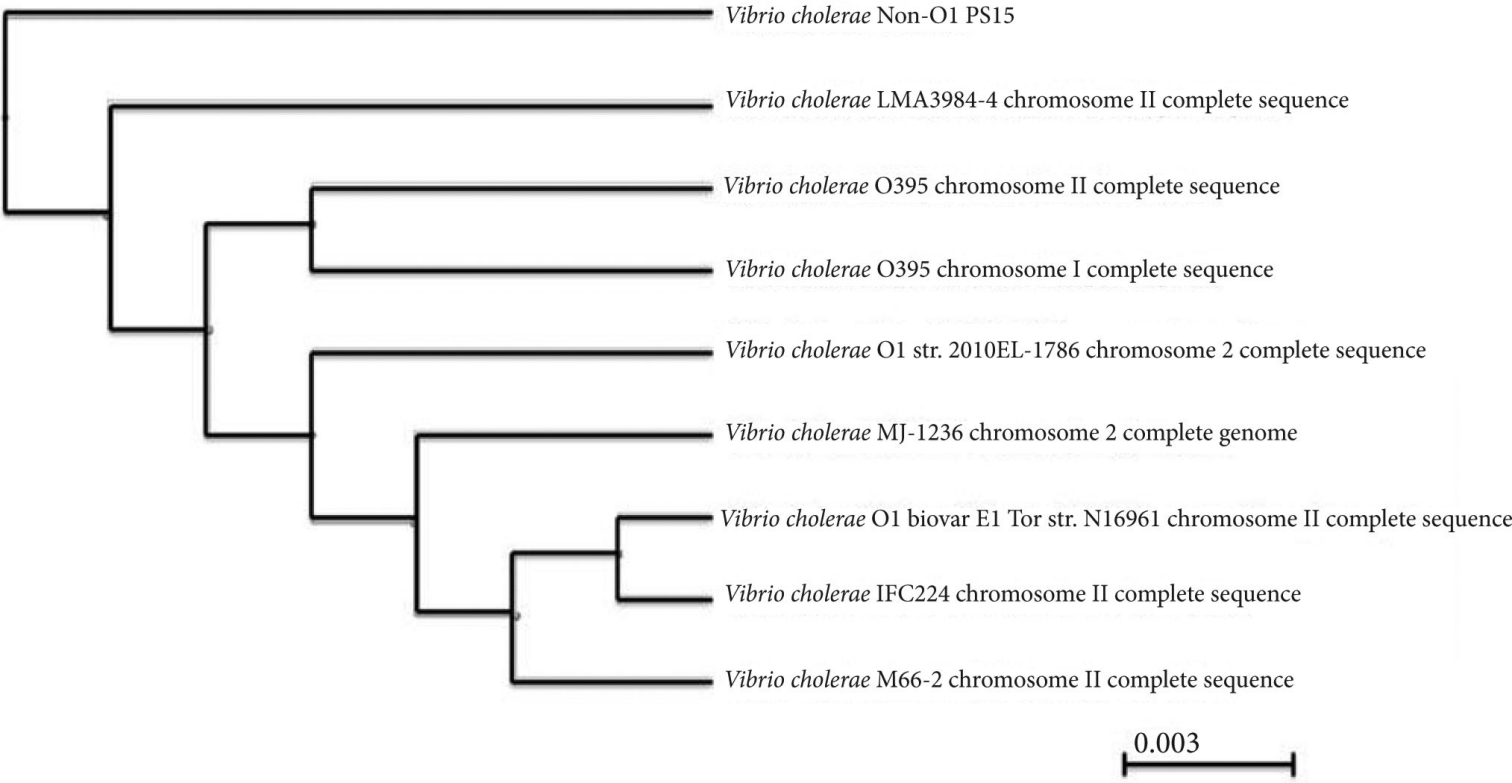
Phylogenetic tree showing comparison between the non-O1 *Vibrio cholerae* PS15 genome and its most closely related microorganisms A phylogenetic tree was generated using BLAST pair wise alignment by the Tree Neighbor Joining method for the DNA sequence of PS15. The distance between the internal node for a subtree or alignment is 0.003.The accession numbers for the sequences selected are as follows: *V. cholerae* non-O1 PS15 (AIJR00000000), *V. cholerae* LMA3984-4 chromosome II complete sequence (NC_017269.1), *V. cholerae* O395 chromosome II complete sequence (NC_012583.1), *V. cholerae* O395 chromosome I complete sequence (NC_009456.1), *V. cholerae* O1 str. 2010EL-1786 chromosome 2 complete sequence (NC_016446.1), *V. cholerae* MJ-1236 chromosome 2 complete genome (NC_012667.1), *V. cholerae* O1 biovar El Tor str. N16961 chromosome II complete sequence (NC_002506.1), *V. cholerae* IEC224 chromosome II complete sequence (NC_016945.1), and *V. cholerae* M66-2 chromosome II complete sequence (NC_012580.1).

The non-O1 *V. cholerae* PS15 genome sequence [28] (GenBank Accession no. AIJR00000000) was analyzed using BLAST [34] in order to generate phylogenetic trees harboring genomes of closely related organisms and virulence factors of the O1 serotypes. The BLAST pair wise alignment using Tree Neighbor Joining method [35] was used to compare the genome of PS15 to other complete *Vibrio* genome sequences in the database and is represented in **Figure 1**.

### CGView

The CGView server was used for comparative genome analysis [36]. A graphical circular genome map was constructed using CGView by BLAST analysis of the DNA sequence of *V. cholerae* non- O1 PS15 (3,910,387 base pairs) with the complete DNA sequence of *V. cholerae* El Tor N16961 (4,033,460 base pairs) [28,30].

## Results

### The genome of non-O1 *V. cholerae* PS15 is distantly related to O1 *V. cholerae* genomes

We previously determined the whole genome sequence of a non-toxigenic, non-O1 *V. cholerae* isolate from Puget Sound, strain PS15 [28]. It had been shown that genomes of toxigenic O1 *V. cholerae* bacteria were highly related [30], possibly implying that non-O1 genomes would be more distantly related. We tested this prediction by comparing non-O1 *V. cholerae* PS15 with other microorganisms by constructing a phylogenetic tree using BLAST pair-wise alignment in order to represent genomes that are most closely related to *V. cholerae* non-O1 PS15 and to establish relatedness of PS15 to these microorganisms (**Figure 1**). Although the non-O1 *V. cholerae* PS15 genome sequence is most closely related to those of *V. cholerae* LMA 3984-4, O395, O1 strains 2010EL-1786, MJ-1236, O1 biovar El Tor strain N16961, IEC224, and M66-2, the non-O1 *V. cholerae* PS15 strain is, nonetheless, the most distantly related member within this cluster.

### Tentative chromosome assignment in non-toxigenic, non-O1 *V. cholerae* PS15

**Figure 2:**
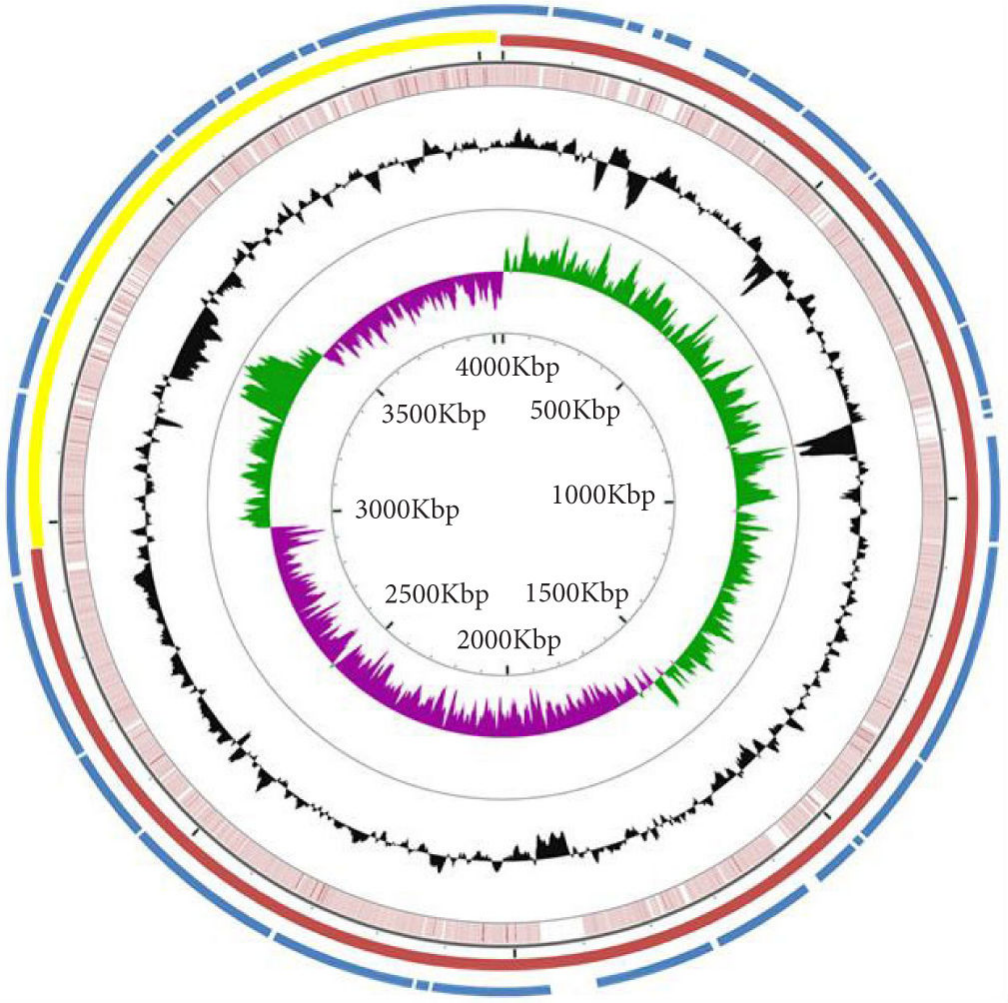
Circular genome map comparing *Vibrio cholerae* non-O1 PS15 with O1 *V. cholerae* N16961 BLAST was performed on the DNA sequence of the *V. cholerae* non-O1 PS15 (3,910,387 base pairs) with *V. cholerae* El Tor N16961 (4,033,460 base pairs) using CGView server to yield a structural representation of both genomes. The FASTA sequences of the genomes were used to generate the graphical circular genome map, and the Global Blast Settings or parameters selected were as follows: query split size=50,000, and overlap split size=0; Blast 1: non-O1 *V. cholerae* PS15, and blastn expect=0.1. The outermost circle in blue represents the entire genome of non-O1 *V. cholerae* PS15, and PS15 gaps in the genome alignment are indicated in white. In the next inner circle, the red and yellow color coded regions represent chromosomes 1 and 2, respectively, of *V. cholerae* N16961. The third inner circle in pink shows matching nucleotide base pairs representing the BLAST analysis for *V. cholerae* N16961 and *V. cholerae* PS15. The fourth inner circle represents the total G+C content color coded in black. In the fifthh inner circle, the region in green represents the G+C content of the forward strand, and the region in purple represents the G+C content of the reverse strand. The innermost circle represents the base pair numbers, where kbp stands for kilo base pairs.

Since the two chromosomes of the toxigenic O1 *V. cholerae* strain N16961 were elucidated [30], we predicted that genomic sequence alignment with the non-toxigenic, non-O1 *V. cholerae* strain PS15 would implicate chromosome assignment in this bacterium as well. A circular genome representation was generated using the CGView server to plot the structural genome arrangement with BLAST analysis of the non-O1 *V. cholerae* PS15 genome with that of the O1 *V. cholerae* N16961 using their respective genomic nucleotide sequences in a FASTA format (**Figure 2**). Using the genome sequence data from *V. cholerae* N16961 to compare with the genome of *V. cholerae* PS15, chromosomes 1 and 2 were implicated for the non-toxigenic PS15 strain and are shown in **Figure 2**.

### The majority of genes in the O1 N16961 and non-O1 PS15 *V. cholerae* genomes are shared

We have shown above that although the non-O1 *V. cholerae* PS15 genome is distantly related to the genomes of toxigenic O1 *V. cholerae*, the PS15 genome is still closely related to genomes of the *Vibrio* genus. This implies a striking similarity between the non-O1 and O1 genomes, specifically regarding the commonalities within the gene space. To test this, we used RAST Seed Viewer and UniProt to compare the genome sequences of O1 *V. cholerae* N16961 and non-O1 *V. cholerae* PS15, the general features of which are shown in **Table 1**. The O1 and non-O1 *V. cholerae* genomes shared 766 genes (open reading frames) that are predicted to code for proteins within functional categories pertaining to virulence, disease, defense, membrane transport, phages, prophages, transposable elements, plasmids, DNA metabolism, dormancy, sporulation and regulons. Interestingly, when compared to the N16961 genome, the *V. cholerae* PS15 genome appears to be truncated sporadically throughout by approximately 120 kbp (**Table 1** and **Figure 2**). In **Table 2** we listed 58 of 766 genes that share 98% identity between both genomes. The remaining genes are listed in **Supplement Table S1**. Even though non-O1 *V. cholerae* PS15 is believed to be non-pathogenic compared to the known virulent O1 *V. cholerae* N16961 strain, their genomes shared 90 genes in common that code for functions pertaining to virulence, disease and defense. Some of these genes included accessory colonization factor (*acfD*), TCP pilus virulence regulatory protein (*tcpN*), toxin coregulated pilus biosynthesis protein E (*tcpE*), TCP pilus virulence regulatory protein (*toxT*) and accessory colonization factor (*acfC*). In addition to these virulence-associated genes, both genomes shared 287 genes encoding functional properties in the DNA metabolism category, 8 genes encoding proteins for dormancy and sporulation, 366 genes encoding membrane transporters, 12 genes in the categories of phages, prophages, transposable elements and plasmids, and 3 genes pertaining to regulons. Among these shared genomic elements encoding membrane transporters are genes known to express multidrug resistance efflux pumps, including AcrA of the RND superfamily [37], SugE of the SMR superfamily [38], and NorM of the MATE superfamily [39].

### Genes present in O1 *V. cholerae* N16961 genome and absent in the non-O1 PS15 genome

The pathogenicity of the O1 *V. cholerae* serotypes suggests that they harbor genomic elements that confer virulence. For instance, the cholera toxin of toxigenic *V. cholerae* strains is the primary virulence factor in endemic and pandemic cholera cases [40]. Thus, in order to establish the association between presence of virulence-encoding genomic elements and pathogenicity, we compared the functional determinants between both PS15 and N16961 genomes. Our analysis revealed that of the 619 genes absent in the non-O1 *V. cholerae* PS15 genome [29], 56 of these genes, when compared to O1 *V. cholerae* N16961, are in the categories including virulence, disease and defense, membrane transport, DNA metabolism, dormancy and sporulation (**Table 3**). The virulence genes which were present in O1 serotypes but largely absent in the non-O1 strains, including the PS15 strain, include the accessory cholera enterotoxin (*ace*), the cholera enterotoxin subunit B (*ctxB*), the cholera enterotoxin subunit A (*ctxA*), and the zona occludens toxin (*zot*). Comparison of the predicted proteins encoded of both PS15 and N16961 genomes using UniProt revealed the absence of other virulence genes in PS15, which include genes predicted to encode accessory colonization factors A and B (*acfA* and *acfB*), and the genes encoding VceA and VceB proteins shown to confer resistance to antimicrobial agents (**Table 3**) [41]. Notably, the gene demonstrated to confer multidrug resistance and encoding a drug efflux pump, EmrD-3, of the MFS is present in N16961 but absent from the non-O1 *V. cholerae* PS15 genome [17,21].

**Figure 3:**
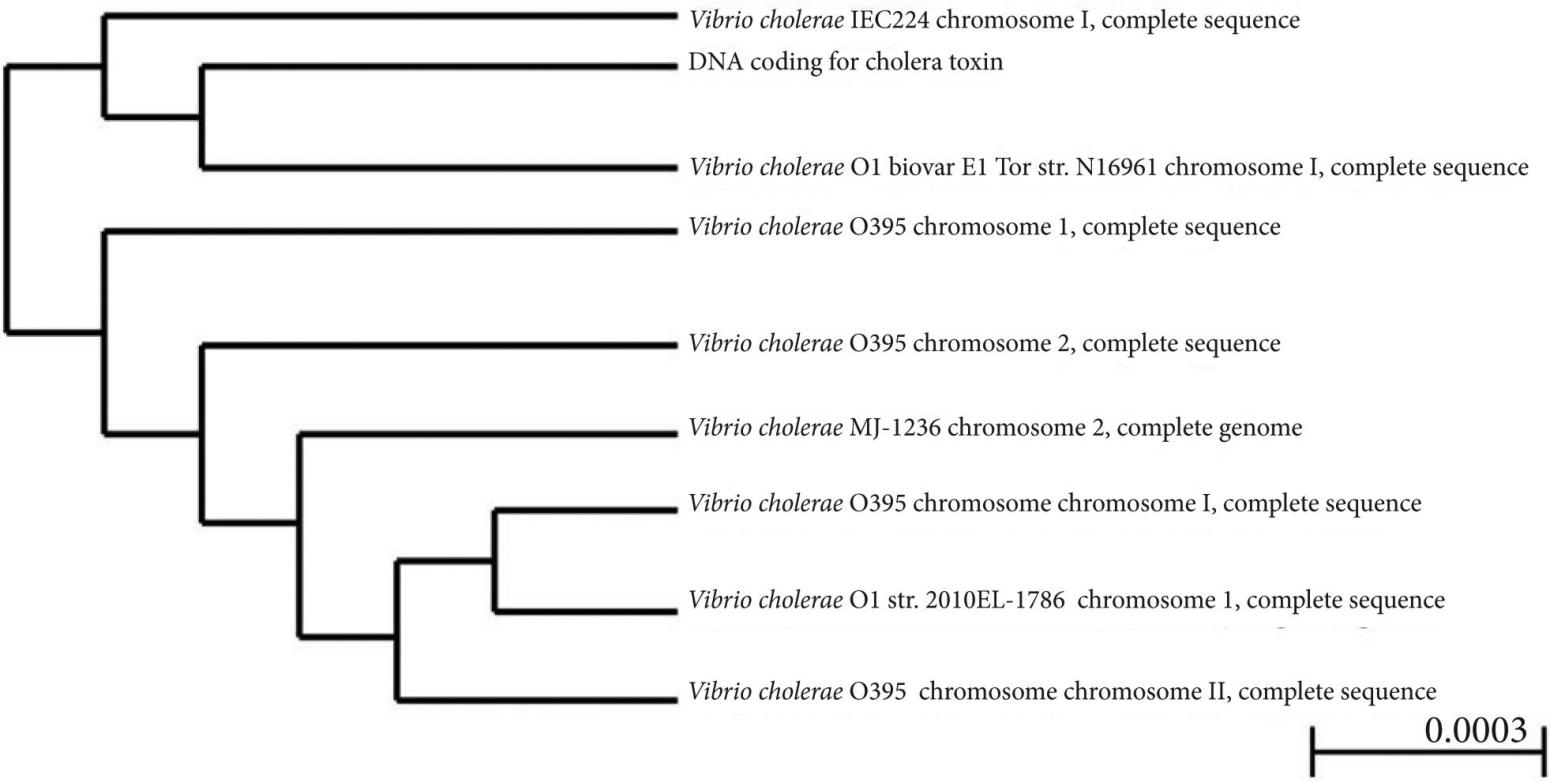
Phylogenetic tree for genomes sharing the cholera toxin A phylogenetic tree representing virulence of *V. cholerae* O1 due to the presence of cholera toxin was produced by the Neighbor Joining Tree method, using BLAST pair wise alignment. Distance between internal node for a subtree or alignment is 0.0003. The distance tree of the result was generated by BLAST for sequences that share DNA encoding the cholera toxin. The accession numbers for the sequences selected are as follows: DNA coding the cholera toxin: GenBank: E00132.1, *V. cholerae* IEC224 chromosome I, complete sequence (NC_016944.1), *V. cholerae* O1 biovar El Tor str. N16961 chromosome I, complete sequence (NC_002505.1), *V. cholerae* O395 chromosome 1, complete sequence (NC_009456.1), *V. cholerae* O395 chromosome 2, complete sequence (NC_009457.1), *V. cholerae* MJ-1236 chromosome 2, complete genome (NC_012667.1), *V. cholerae* O395 chromosome I, complete sequence (NC_012582.1), *V. cholerae* O1 str. 2010EL-1786 chromosome 1, complete sequence (NC_016445.1), and *V. cholerae* O395 chromosome II, complete sequence (NC_012583.1).

A phylogenetic tree, which was generated by BLAST for bacterial genomes that share the cholera toxin, indicated the absence of the cholera toxin gene in the non-O1 *V. cholerae* PS15 bacterium (**Figure 3**). The most closely-related microorganisms that shared the DNA encoding the cholera toxin include *V. cholerae* IEC224, O1 biovar El Tor strain N16961, O395, MJ-1236 and the O1 strain 2010EL-1786.

Other genes that were absent in non-O1 *V. cholerae* genome but present in O1, include genes that encode glycerolipid and glycerophospholipid metabolism, and genes that code for VPI [25] (**Table 3**). Additional genes that are absent in non-O1 *V. cholerae* PS15 include those coding for the Rst operon essential for the synthesis of phage related replication protein (RstA), phage related integrase (RstB), phage related antirepressor (RstC), phage related transcriptional repressor (RstR) [24], and sulfur metabolism. Other genes that are found in O1 *V. cholerae* but absent in non-O1 include those coding for TsaE, a protein required for the synthesis of threonylcarbamoyladenosine in the presence of tRNA [42].

### Genes present in the non-O1 *V. cholerae* PS15 genome and absent in the O1 N16961 genome

Because the non-O1 *V. cholerae* PS15 environmental isolate is considered to be non-toxigenic [31,43], this implies that genes unique to this microorganism, compared to the toxigenic N16961 bacterium, possibly encode non-virulent functions. To test this hypothesis, we performed a function based genome comparison using RAST and UniProt for PS15 and N16961. This comparative analysis revealed that 113 genes were excluded in N16961 but present within the PS15 genome (**Table 4**). The three known genes (characterized) that are present in PS15, but absent in N16961, include the oligopeptide ABC transporter called periplasmic oligopeptide-binding protein (OppA) [44], a protein-export membrane protein (SecF) [45], and the UvrABC system protein A (*uvrA*) [46], all of which belong to the membrane transport category. Remaining genes annotated as uncharacterized hypothetical proteins as per UniProt are surprisingly predicted to code for proteins involved in functions related to virulence, pathogenesis, defense, solute transport, and DNA metabolism (**Table 4**).

## Conclusions

Upon comparison of the non-O1 *V. cholerae* PS15 genome, a non-toxigenic strain, to that of an O1 *V. cholerae* N16961, a toxigenic strain, we found that of the 619 missing genes, 56 of these missing genomic elements encode dormancy, sporulation, ribosome modulation in persister cells, lipid metabolism, phage infection, nucleoside metabolism, and sulfur metabolism which in turn is essential for biosynthesis of amino acids, vitamins and prosthetic groups [43]. As non-O1 *V. cholerae* lacks genes coding for metabolism of sulfur, the non-O1 serotype is predicted to be unable to convert naturally available sulfur to sulfide, which could then be incorporated into various sulfur containing metabolites. Sulfur is critical for the biosynthesis of many important compounds like amino acids (cysteine and methionine), vitamins (biotin, thiamin), and prosthetic groups (Fe-S clusters) [43]. These genetic elements and their putative gene products represent novel and promising targets for modulation of gene expression or activity and therapeutic efforts [47], in order to effectively reduce conditions that foster virulence and dissemination of *V. cholerae* pathogens through populations. These determinants, therefore, clearly also warrant further studies in order to elucidate the complete molecular mechanisms of pathogenesis in cholera infections.

Not surprisingly, also among the 56 missing genes in the non-O1 PS15 genome are those that are known to confer virulence, such as the cholera toxin [40], colonization factors [48], and antimicrobial resistance mechanisms [16]. We thus confirm that the genes encoding the cholera toxin are absent from the genome of the non-toxigenic *V. cholerae* PS15. We confirm, however, the presence of other genes predicted to encode distinct toxins and colonization factors, as previously shown for the non-O1 *V. cholerae* strain NRT36S [49]. This latter study and our findings here are consistent with previous work demonstrating that aquatic environments are reservoirs for O1 and non-O1 *V. cholerae* [50], predicting that such environments allow genetic exchange between unrelated strains. In order to gain valuable insights into enhancing chemotherapeutic efficacy against cholera, it is imperative to study and gain understanding into the modes of action of the toxicity-inducing factors combined with other antibacterial resistance factors in toxigenic *V. cholerae* [51].

Interestingly, we found that the genome of the nontoxigenic *V. cholerae* PS15 strain harbors genes absent from the genome of its toxigenic counterpart, N16961. Such determinants mainly include still uncharacterized genetic elements that are predicted to encode proteins that confer virulence, disease, defense, membrane solute transport and DNA metabolism, suggesting that PS15 may be pathogenic to organisms excluding humans, perhaps in environments such as estuary waters [52,53]. Among the genetic determinants unique to PS15 that have been experimentally characterized include OppA, an oligopeptide primary active transporter [44], and SecF, a protein exporter [12]. We propose that these unique genetic elements represent good targets for future development of new therapies against *V. cholerae* infections in animals other than humans.

The genome of non-O1 *V. cholerae* PS15 shares >97% identity with El Tor O1 biovar *V. cholerae* strain N16961, as per BLAST analysis at the nucleotide level. Based on the alignment of the non-O1 PS15 genome with that of O1 N16961, chromosomes 1 and 2 were assigned to the PS15 genome (**Figure 2**). This tentative chromosome assignment will require confirmation with additional experimental work. Even though the genomes of both strains are highly similar to each other, the non-O1 PS15 microorganism is considered to be non-pathogenic, compared to the O1 N16961 strain, possibly due to the absence of the cholera toxin in PS15, which is responsible for endemic and pandemic diseases [54]. More recent genomic analysis, however, has demonstrated that other genetic elements are also critical for conferring pathogenesis such as genes coding for housekeeping, homeostasis, metabolism, energy generation, and antimicrobial resistance-type functions [55]. Our phylogenetic and genome comparison analyses between the toxigenic and non-toxigenic *V. cholerae* microorganisms support both of these contentions. Further work with additional variants, such as atypical El Tor [56], NRT36S [49], and CT-producing non-O1 strains [57], will be necessary to definitively gain a complete picture of the relationships between pathogenic versus non-pathogenic *V. cholerae*.

Remarkably, we found that both of the toxigenic and non-toxigenic *V. cholerae* strains harbor a variety of genes that have previously been demonstrated to confer multidrug resistance via active drug efflux pump systems, such as AcrAB, NorM / VcmA, SugE, and VcaM [58]. All six RND transporters in *V. cholerae* N16961 have been studied physiologically [59], and our data showed that *V. cholerae* PS15 was missing only one of these pumps, called VexA. Additionally, we found a shared but uncharacterized genetic element, *VC_A0083* in the toxigenic strain and *OSU_1537* in the non-toxigenic strain, tentatively called multidrug resistance protein D and predicted to encode an MFS drug efflux pump. These multidrug resistance mechanisms may be important because of their potential selection and maintenance in environments containing antimicrobial agents, their genetic mobility to other microorganisms, and dissemination within populations [60-64].

We conclude that the study and comparison of the genomic sequences between pathogens and their non-virulent counterparts will help discover genes encoding both the classical virulence factors and those encoding novel virulence factors. Future work will focus on the study of solute transport and antibacterial resistance mechanisms of *V. cholerae* pathogenic strains and on the identification of novel housekeeping genes which may be equally significant in contributing towards the microorganisms’ pathogenicity [17,65,66].

## Supplementary Material

Supplemental Table S1

## Figures and Tables

**Table 1:** Bacterial strains and protein encoding genes.

Strain	*V. cholerae N16961*	*V. cholerae PS15*
Length (bp)	4,033,460	3,910,387
G+C content (%)	47.7 (chromosome 1)	47.55
	46.9 (chromosome 2)	--
Number of protein-coding genes with assigned function	424	272
Number of hypothetical	398	607
Total number of genes	822	879

Comparison of the general features for O1 *V. cholerae* N16961 and non-O1 *V. cholerae* PS15 contrasting the base pair lengths (bp), GC content, and genes associated with the biological or physiological categories such as virulence, disease, defense, membrane transport, DNA metabolism, regulons, dormancy, sporulation, phages, prophages, transposable elements and plasmids.

**Table 2:** Genes shared between genomes of non-O1 *Vibrio cholerae* PS15 and O1 *V. cholerae* N16961.

Description	Abbreviation N16961	Accession N16961	Abbreviation PS15	Accession PS15
DNA gyrase inhibitor YacG^*^	*yacG*	Q9KPE1	*yacG*	L1QXA7
Transcription elongation factor GreB^*^	*greB*	Q9KNL7	*greA*	L1R2F8
Autoinducer 2 sensor kinase/phosphatase LuxQ^*^	*luxQ*	Q9KLK7	*OSU_2901*	L1QTW3
Putative uncharacterized protein VC0929^*^	*VC0929*	Q9KTH3	*OSU_1349*	L1QYD5
Putative uncharacterized protein^*^	*VC_A0118*	Q9KN47	*OSU_1575*	L1QXZ3
Release factor glutamine methyltransferase^†^	*prmC*	Q9KQ26	*prmC*	L1QU18
Probable potassium transport system protein kup^†^	*kup*	Q9KM59	*kup*	L1QTS9
Electron transport complex protein RnfC^†^	*rnfC*	Q9KT88	*rnfC*	L1QUE4
Probable oxaloacetate decarboxylase gamma chain 2^†^	*oadG2*	Q9KTU4	*oadG*	L1R348
Thiamine import ATP-binding protein ThiQ^†^	*thiQ*	Q9KP42	*OSU_1092*	L1QYX4
Vitamin B12 import ATP-binding protein BtuD^†^	*btuD*	Q9KSL1	*btuD*	L1R0X6
Copper-exporting P-type ATPase A^†^	*copA*	Q9KPZ7	*OSU_0952*	L1QZQ5
Putative fluoride ion transporter CrcB^†^	*crcB*	Q9KVS9	*crcB*	L1QWW5
MSHA biogenesis protein MshG^†^	*VC_0406*	Q9KUV6	*OSU_1460*	L1QXZ4
MSHA biogenesis protein MshH^†^	*VC_0398*	Q9KUW1	*OSU_1452*	L1QYV1
Sigma-54 dependent transcriptional regulator^†^	*VC_1817*	Q9KR30	*OSU_1624*	L1QXJ1
Transport ATP-binding protein CydD^†^	*VC_1181*	Q9KSS5	*OSU_2736*	L1QUK9
Transport ATP-binding protein CydC^†^	*VC_1180*	Q9KSS6	*OSU_2737*	L1QVL8
Amino acid ABC transporter, permease proteint	*VC_A1040*	Q9KKR2	*OSU_1389*	L1QY57
Peptide ABC transporter, permease protein, putative^†^	*VC_A0590*	Q9KLZ9	*OSU_0554*	L1R1Z6
Multidrug transporter, putative^†^	*VC_1391*	Q9KS68	*OSU_2315*	L1QVM0
Thiamin ABC transporter, periplasmic thiamin-binding protein^†^	*VC_2539*	Q9KP40	*OSU_1094*	L1QYM0
Cbb3-type cytochrome c oxidase subunit^†^	*VC_1439*	Q9KS22	*OSU_0068*	L1R226
Benzoate transport protein^†^	*VC_1970*	Q9KQM8	*OSU_3112*	L1QUT1
ABC transporter, permease protein^†^	*VC_A1099*	Q9KKK5	*OSU_2952*	L1QTR7
Transporter, LysE family^†^	*VC_1712*	Q9KRD0	*OSU_1426*	L1QZ42
ABC-type multidrug transport system, permease component^†^	*VC_0590*	Q9KUD2	*OSU_0545*	L1R0T8
Proton/glutamate symport protein / Sodium/glutamate symport protein^†^	*VC_A0088*	Q9KN77	*OSU_1542*	L1QY02
NADH dehydrogenase, putative^†^	*VC_1581*	Q9KRQ5	*OSU_1036*	L1R051
Na^+^/H^+^ antiporter, putative^†^	*VC_0389*	Q9KUX0	*OSU_1443*	L1QY90
Cytochrome b561, putative^†^	*VC_A0249*	Q9KMS1	*OSU_0494*	L1R0V0
Osmosensitive K^+^ channel histidine kinase KdpD/ Sensor histidine kinase^†^	*VC_A0531*	Q9KM57	*OSU_3424*	L1QSJ3
Xanthine/uracil permease family proteint	*VC_2712*	Q9KNM0	*OSU_0044*	L1R323
DNA polymerase I/DNA polymerase II/ DNA polymerase IV^§^	*dinB*	Q9KPS5	*dinB*	L1R1B3
Nuclease SbcCD subunit C^§^	*sbcC*	Q9KM67	*OSU_3415*	L1QTC3
Deoxyribodipyrimidine photo-lyase^§^	*phrA*	Q9KNA8	*OSU_1512*	L1QXV4
Tyrosine--tRNA ligase 1^§^	*tyrS1*	Q9KUQ0	*tyrS*	L1QY24
Formate–tetrahydrofolate ligase^§^	*fhs*	Q9KLX7	*fhs*	L1R108
Valine--tRNA ligase^§^	*valS*	Q9KP73	*valS*	L1QXG6
ADP-L-glycero-D-manno-heptose-6-epimerase^§^	*hldD*	Q06963	*hldD*	L1QT14
Ferrochelatase^§^	*hemH*	Q9KTB6	*hemH*	L1QUK6
Tetraacyldisaccharide 4′-kinase^§^	*lpxK*	Q9KQX0	*lpxK*	L1QYU9
7-carboxy-7-deazaguanine synthase^§^	*queE*	Q9KS94	*queE*	L1QVP2
A/G-specific adenine glycosylase^§^	*VC_0452*	Q9KUR3	*OSU_1912*	L1QX56
Exodeoxyribonuclease V alpha chain^§^	*VC_2319*	Q9KPP7	*OSU_0379*	L1R140
Exodeoxyribonuclease V beta chain/Exodeoxyribonuclease III^§^	*VC_2320*	Q9KPP6	*OSU_0378*	L1R0Z3
Exodeoxyribonuclease V gamma chain^§^	*VC_2322*	Q9KPP4	*OSU_0376*	L1R185
DNA helicase IV^§^	*VC_A0204*	Q9KMW4	*OSU_3456*	L1QTP1
Putative phosphatase YqaB^§^	*VC_A0662*	Q9KLS9	*OSU_0250*	L1R2A2
Non-canonical purine NTP phosphatase^§^	*VC_0702*	Q9KU27	*OSU_3053*	L1QU16
Non-canonical purine NTP pyrophosphatase^§^	*VC_0456*	Q9KUQ9	*OSU_1907*	L1QX52
Putative quercetin 2,3-dioxygenase VC_A0969^§^	*VC_A0969*	Q9KKY1	*OSU_1671*	L1QYQ8
Aldose 1-epimerase^§^	*VC_1594*	Q9KRP2	*OSU_1049*	L1QZA8
Dihydrofolate reductase^§^	*VC_0440*	Q9KUS5	*OSU_1494*	L1QZC9
Cbb3-type cytochrome c oxidase subunit^§^	*VC_1439*	Q9KS22	*OSU_0068*	L1R226
Molybdopterin-guanine dinucleotide biosynthesis protein MobA^✖^	*mobA*	Q9KRV8	*mobA*	L1QZN1
Molybdopterin-guanine dinucleotide biosynthesis protein MobB^✖^	*VC_1527*	Q9KRV7	*OSU_0936*	L1QZT6
DamX-related protein^∥^	*VC_2627*	Q9KNV3	*OSU_1180*	L1QZ10

The table represents a list of genes present in both of the genomes in which the genes share 98 % identity. Included in this table are genes predicted to code for categories including virulence, disease and defense, membrane transport, DNA metabolism, dormancy and sporulation and regulons. In the table, the first column includes protein designations and descriptions. The second and fourth columns represent abbreviated gene identifications; the third and fifth represent accession numbers for the listed genes.

* Proteins with the symbol have putative functions in virulence, disease and defense.

† Proteins with and symbols represent proteins that have functions in membrane transport and DNA metabolism categories, respectively.

§ Proteins with and symbols represent proteins that have functions in membrane transport and DNA metabolism categories, respectively.

∥ Proteins with the symbol have functions within dormancy and sporulation categories, and include proteins that are part of regulons.

✖ Proteins with the symbol have functions within dormancy and sporulation categories, and include proteins that are part of regulons.

**Table 3:** Genes absent in non-O1 *Vibrio cholerae* genome and present in O1 *V. cholerae* genome.

Description	Abbreviation N16961	Accession N16961
Accessory cholera enterotoxin^*^	*ace*	P38441
Cholera enterotoxin subunit B^*^	*ctxB*	P01556
Cholera enterotoxin subunit A^*^	*ctxA*	P01555
Zona occludens toxin^*^	*zot*	P38442
Toxin coregulated pilus biosynthesis protein F^*^	*tcpF*	P0C6Q5
Toxin-coregulated pilus biosynthesis protein P^†^	*tcpP*	Q7BGC9
Toxin coregulated pilus biosynthesis protein I^†^	*tcpI*	P0C6D8
Toxin coregulated pilus biosynthesis protein H^†^	*tcpH*	P29489
Toxin coregulated pilus biosynthesis protein B^†^	*tcpB*	P23476
Toxin coregulated pilus biosynthesis protein E^†^	*tcpE*	P0C6C9
Transcriptional activator protein NhaR^†^	*nhaR*	P52692
Outer membrane lipoprotein blc^†^	*blc*	Q08790
ATP synthase protein I^†^	*atpI*	Q9KNG8
Type 4 prepilin-like proteins leader peptide-processing enzyme^§^	*tcpJ*	P0C6D9
N5-carboxyaminoimidazole ribonucleotide synthase^§^	*purK*	Q9KVT8
Coproporphyrinogen-III oxidase^§^	*hemF*	Q9KVT4
Aldehyde dehydrogenase^§^	*aldA*	P0C6D7
Putative N-acetylmannosamine-6-phosphate 2-epimerase^§^	*nanE*	Q9KR62
N-acetylmannosamine kinase^§^	*nanK*	Q9KR61
N-acetylneuraminate epimerase^§^	*nanM*	Q9KR69
N5-carboxyaminoimidazole ribonucleotide mutase^§^	*purE*	Q9KVT7
Ribosome modulation factor^∥^	*Rmf*	Q9KRZ9
Accessory colonization factor AcfA^*^	*VC_0844*	H9L4S5
Accessory colonization factor AcfB^*^	*VC_0840*	Q9KTQ7
TagE protein^*^	*VC_A1043*	Q9KKQ9
TagE protein^*^	*VC_0843*	H9L4P5
Uncharacterized protein VC_1460^*^	*VC_1460*	P38443
Fimbrial biogenesis and twitching motility protein, putative^†^	*VC_1612*	Q9KRM4
Type IV pilin, putative^†^	*VC_0858*	Q9KTP3
Fimbrial protein^†^	*VC_2423*	Q9KPE5
Fimbrial assembly protein^†^	*VC_2630*	Q9KNV0
Putative uncharacterized protein^†^	*VC_1703*	Q9KRD9
RTX toxin transporter^†^	*VC_1448*	Q9KS14
Uncharacterized protein similar to VCA0109^†^	*VC_A0109*	Q9KN56
C4-dicarboxylate transport protein DctQ, putative^†^	*VC_1928*	Q9KQS0
Trk system potassium uptake protein^†^	*VC_0042*	Q9KVU7
PTS system, cellobiose-specific IIC component^†^	*VC_1282*	Q9KSH4
Multidrug resistance protein VceB^†^	*VC_1411*	Q9KS49
Iron(III) compound receptor^†^	*VC_0200*	Q9KVE6
Sugar transporter family protein^†^	*VC_A0669*	Q9KLS2
Potassium uptake protein TrkA^†^	*VC_0043*	Q9KVU6
Multidrug resistance protein, putative^†^	*VC_1409*	Q9KS51
Sodium/solute symporter^†^	*VC_A0667*	Q9KLS4
C4-dicarboxylate-binding periplasmic protein^†^	*VC_1779*	Q9KR64
Multidrug resistance protein D^†^	*VC_A0214*	Q9KMV4
Multidrug resistance protein D^†^	*VC_A0267*	Q9KMQ3
PTS system, N-acetylglucosamine-specific IIABC component^†^	*VC_0995*	Q9KTA8
Lipopolysaccharide/O-antigen transport protein^†^	*VC_0246*	Q9KVA3
Iron(III) ABC transporter, permease protein^†^	*VC_0203*	Q9KVE3
Putative uncharacterized protein^†^	*VC_A0716*	Q9KLM7
Multidrug resistance protein VceA^†^	*VC_1410*	Q9KS50
Putative uncharacterized protein^†^	*VC_A0355*	Q9KMJ3
Helicase, putative^§^	*VC_1760*	Q9KR83
DNA-damage-inducible protein J^§^	*VC_A0324*	Q9KML3
N-acetylglucosamine-6-phosphate deacetylase^§^	*VC_1783*	Q9KR60
Sigma-54 modulation protein, putative^∥^	*VC_2530*	H9L4N9

Included in this table are genetic elements that are absent in the non-Ol genome but present in the O1 genome, which have putative functions in virulence, disease and defense, membrane transport, DNA metabolism and dormancy and sporulation. In the table, the first column includes gene descriptions as per UniProt. Second and fourth columns represent abbreviated gene identification; the third and fifth columns represent accession numbers for the listed genes.

* The symbol denotes proteins that have functions in virulence, disease and defense.

† The symbol includes proteins that are putative membrane transporters.

§ Symbols and include proteins that have putative functions in DNA metabolism and dormancy/sporulation categories, respectively.

∥ Symbols and include proteins that have putative functions in DNA metabolism and dormancy/sporulation categories, respectively.

**Table 4:** Genes present in non-O1 *Vibrio cholerae* genome but absent in O1 *V. cholerae.*

Description	Abbreviation PS15	Accession PS15
Oligopeptide ABC transporter, periplasmic oligopeptide-binding protein OppA^†^	*oppA*	L1QVD3
Protein-export membrane protein SecF^†^	*secF*	L1QTX8
UvrABC system protein A^†^	*uvrA*	L1QY95
CopG protein^*^	*OSU_0951*	L1R0A8
Cytochrome c heme lyase subunit CcmF^*^	*OSU_1000*	L1QZI8
Cytochrome c heme lyase subunit CcmH^*^	*OSU_1003*	L1R0Q0
Cytochrome c heme lyase subunit CcmL^*^	*OSU_1002*	L1QZP2
Multi antimicrobial extrusion protein (Na^+^/drug antiporter) VcrM^*^	*OSU_3002*	L1QUH8
Multidrug and toxin extrusion (MATE) family efflux pump YdhE/NorM^*^	*OSU_0958*	L1R0S0
Multidrug efflux pump component MtrF^*^	*OSU_0277*	L1R1D8
Putative queD like protein^*^	*OSU_1874*	L1QXS7
Type IIA topoisomerase, B subunit^*^	*OSU_0552*	L1R212
Arsenical resistance operon repressor^*^	*OSU_0350*	L1R2G7
Arsenical-resistance protein ACR3^*^	*OSU_0349*	L1R1D6
Copper-sensing two-component system response regulator CusR^*^	*OSU_3536*	L1QTJ3
DNA-binding heavy metal response regulator^*^	*OSU_2602*	L1QVH5
Multidrug resistance transporter, Bcr/CflA family^*^	*OSU_2210*	L1QWA2
MFS family multidrug transport protein, bicyclomycin resistance protein^*^	*OSU_0873*	L1QZR8
Macrolide export ATP-binding/permease protein MacB^*^	*OSU_3185*	L1QT51
P pilus assembly/Cpx signaling pathway, periplasmic inhibitor/zinc-resistance protein^*^	*OSU_1241*	L1QZY1
Cobalt-zinc-cadmium resistance protein^*^	*OSU_1240*	L1QZ70
Cobalt-zinc-cadmium resistance protein^*^	*OSU_2129*	L1QWD3
Cobalt-zinc-cadmium resistance protein CzcD^*^	*OSU_1105*	L1QYT4
Cytolysin and hemolysin, HlyA, Pore-forming toxin^*^	*OSU_0766*	L1R001
Metalloprotease, containing putative zinc-binding domain^*^	*OSU_0770*	L1R1C4
Translation initiation factor SUIl-related protein^*^	*OSU_0889*	L1R0U9
Transcription initiation factor TFIIIB, Brfl subunit/Transcription initiation factor TFIIB^*^	*OSU_0614*	L1R0L1
ABC-type tungstate transport system, ATP-binding protein^†^	*OSU_0934*	L1R0Z5
ABC-type tungstate transport system, periplasmic binding protein^†^	*OSU_0932*	L1R0H9
Phosphonate ABC transporter phosphate-binding periplasmic component^†^	*OSU_2433*	L1QWK1
AttE component of AttEFGH ABC transport system^†^	*OSU_0877*	L1R138
AttF component of AttEFGH ABC transport system / AttG component of AttEFGH ABC transport system^†^	*OSU_0878*	L1QZS3
Peptide transport periplasmic protein sapA^†^	*OSU_1956*	L1QWN6
Magnesium and cobalt transport protein CorA^†^	*OSU_0364*	L1R1E9
Mg/Co/Ni transporter MgtE / CBS domain containing protein^†^	*OSU_1331*	L1QYJ2
MSHA biogenesis protein MshO^†^	*OSU_1466*	L1QY52
MSHA biogenesis protein MshP^†^	*OSU_1467*	L1QYW4
MSHA biogenesis protein MshQ^†^	*OSU_1468*	L1QYB3
Multimodular transpeptidase-transglycosylase^†^	*OSU_1188*	L1R077
Multimodular transpeptidase-transglycosylase^†^	*OSU_0534*	L1R0P0
Type IV fimbrial biogenesis protein FimT^†^	*OSU_0787*	L1QZX9
Type IV fimbrial biogenesis protein PilV^†^	*OSU_0784*	L1R0R8
Type IV fimbrial biogenesis protein PilW^†^	*OSU_0786*	L1R195
Type IV pilin PilA^†^	*OSU_2009*	L1QWH3
Type IV pilus biogenesis protein PilE^†^	*OSU_0788*	L1R035
Type IV pilus biogenesis protein PilM^†^	*OSU_1187*	L1QZ62
Type IV pilus biogenesis protein PilN^†^	*OSU_1186*	L1QZS4
Type IV pilus biogenesis protein PilO^†^	*OSU_1185*	L1QZ15
Type IV pilus biogenesis protein PilQ^†^	*OSU_1183*	L1R071
Conjugative signal peptidase TrhF^†^	*OSU_2230*	L1QWL7
Conjugative transfer protein s043^†^	*OSU_2245*	L1QWN3
IncF plasmid conjugative transfer pilus assembly protein TraB^†^	*OSU_2239*	L1QVW4
IncF plasmid conjugative transfer pilus assembly protein TraC^†^	*OSU_2232*	L1QX28
IncF plasmid conjugative transfer pilus assembly protein TraE^†^	*OSU_2241*	L1QW42
IncF plasmid conjugative transfer pilus assembly protein TraF^†^	*OSU_0332*	L1R1B9
IncF plasmid conjugative transfer pilus assembly protein TraH^†^	*OSU_0331*	L1R173
IncF plasmid conjugative transfer pilus assembly protein TraK^†^	*OSU_2240*	L1QWM9
IncF plasmid conjugative transfer pilus assembly protein TraL^†^	*OSU_2242*	L1QX38
IncF plasmid conjugative transfer pilus assembly protein TraU^†^	*OSU_2228*	L1QVR6
IncF plasmid conjugative transfer pilus assembly protein TraW^†^	*OSU_2229*	L1QVV2
IncF plasmid conjugative transfer protein TraD^†^	*OSU_2247*	L1QX42
IncF plasmid conjugative transfer protein TraG^†^	*OSU_0330*	L1R2J2
IncF plasmid conjugative transfer protein TraN^†^	*OSU_2227*	L1QX23
Ync^†^	*OSU_2235*	L1QWM2
Ynd^†^	*OSU_2236*	L1QW36
Toxin co-regulated pilus biosynthesis protein E, anchors TcpT to membrane^†^	*OSU_2156*	L1QXB5
T1SS associated transglutaminase-like cysteine proteinase (LapP)^†^	*OSU_2969*	L1QUR2
Membrane-fusion protein^†^	*OSU_2970*	L1QU65
ABC-type bacteriocin/lantibiotic exporter, containing an N-terminal double-glycine peptidase domain^†^	*OSU_2967*	L1QTT3
Outer membrane protein ImpK/VasF, OmpA/MotB domain containing^†^	*OSU_1572*	L1QY45
Protein ImpG/VasA^†^	*OSU_1567*	L1QY39
Type VI secretion lipoprotein/VasD^†^	*OSU_1570*	L1QXY9
Type VI secretion protein VasI^†^	*OSU_1575*	L1QXZ3
Type VI secretion-related protein VasL^†^	*OSU_1578*	L1QZ51
Uncharacterized protein ImpB^†^	*OSU_1564*	L1QXT4
Uncharacterized protein ImpC^†^	*OSU_1565*	L1QXY4
Uncharacterized protein ImpH/VasB^†^	*OSU_1568*	L1QZ45
Uncharacterized protein ImpI/VasC^†^	*OSU_1569*	L1QXT8
Uncharacterized protein ImpJ/VasE^†^	*OSU_1571*	L1QYQ3
VgrG-3 protein^†^	*OSU_1579*	L1QXU5
TRAP transporter solute receptor, TAXI family^†^	*OSU_1483*	L1QYC6
TRAP transporter solute receptor, TAXI family^†^	*OSU_1598*	L1QZ65
TRAP-type uncharacterized transport system, fused permease component^†^	*OSU_1482*	L1QYX7
TRAP-type C4-dicarboxylate transport system, periplasmic component^†^	*OSU_1751*	L1QXA6
Na^+^/ H^+^ antiporter subunit E^†^	*OSU_1606*	L1QYT1
Na^+^/ H^+^ antiporter subunit F^†^	*OSU_1605*	L1QY16
Na^+^/ H^+^ antiporter subunit G^†^	*OSU_1604*	L1QXW5
Di-/tripeptide transporter^†^	*OSU_0457*	L1R0U7
Di/tripeptide permease DtpA^†^	*OSU_2702*	L1QVD6
4-hydroxybenzoyl-CoA thioesterase family active site protein^†^	*OSU_1280*	L1QZ99
MotA/TolQ/ExbB proton channel family protein^†^	*OSU_3208*	L1QUH0
TPR repeat containing exported protein^†^	*OSU_1274*	L1QYK6
TonB system biopolymer transport component/ Chromosome segregation ATPase^†^	*OSU_3207*	L1QTE4
TonB-dependent heme and hemoglobin receptor HutA/ TonB-dependent hemin, ferrichrome receptor^†^	*OSU_0883*	L1QZU0
PTS system, N-acetylglucosamine-specific IIA, IIB, IIC component^†^	*OSU_2709*	L1QVU6
Ferrichrome-iron receptor^†^	*OSU_1805*	L1QX54
Enterobactin receptor VctA^†^	*OSU_0353*	L1R1Z3
Putative divalent cation transport protein^†^	*OSU_1717*	L1QXK6
Tricarboxylate transport protein TctC^†^	*OSU_2307*	L1QVT4
Membrane fusion component of tripartite multidrug resistance system^†^	*OSU_0595*	L1R1A1
AmpG permease^†^	*OSU_2915*	L1QVA5
High-affinity choline uptake protein BetT^†^	*OSU_2908*	L1QUU0
Uncharacterized protein^†^	*OSU_3399*	L1QSI2
Tricarboxylate transport membrane protein TctA EMBL EKY32019^†^	*OSU_2305*	L1QVL0
Uncharacterized protein^†^	*OSU_2298*	L1QWR3
Ca^2+^/H^+^ antiporter^†^	*OSU_1800*	L1QX51
Putative permease^†^	*OSU_2291*	L1QWA8
Transporter, LysE family^†^	*OSU_0512*	L1R248
Error-prone repair protein UmuD^§^	*OSU_2250*	L1QWN9
Error-prone, lesion bypass DNA polymerase V (UmuC)^§^	*OSU_3541*	L1QX48
Error-prone, lesion bypass DNA polymerase V (UmuC)^§^	*OSU_2251*	L1QW51
Type I restriction-modification system, DNA-methyltransferase subunit M^§^	*OSU_2542*	L1QV27
Type I restriction-modification system, specificity subunit S^§^	*OSU_2543*	L1QVX0

Included in this table are genes coding for virulence, disease and defense, membrane transport and DNA metabolism. The first column includes gene descriptions as per UniProt.

* Second and third columns represent abbreviated identifications and accession numbers for the described genes, respectively, includes proteins that have putative functions in the virulence, disease or defense categories

† symbols represent proteins with functions in membrane transport and DNA metabolism categories, respectively.

§ symbols represent proteins with functions in membrane transport and DNA metabolism categories, respectively.
