# Supplementary material for: Comparative genome analysis of non-toxigenic non-O1 versus toxigenic O1 Vibrio cholerae
Source: Genom Discov. Author manuscript; Available in PMC 2015 Feb 24. (PMC4338557; doi:10.7243/2052-7993-2-1)
Supplement: Supplemental Table S1 [file NIHMS658796-supplement-Supplemental_Table_S1.pdf]

## Supplementary Material

## Table S1: Genes shared between genomes of non-O1 *Vibrio cholerae* and O1 *V. cholerae.*

| Description | Abbreviation N16961 | Accession N16961 | Abbreviation PS15 | Accession PS15 | Identity % |
|---|---|---|---|---|---|
| Accessory colonization factor AcfD* | *acfD* | P0C6F0 | *OSU_1709* | L1QXF4 | 66 |
| TCP pilus virulence regulatory protein* | *tcpN* | P0C6D6 | *OSU_2153* | L1QW48 | 28 |
| Toxin coregulated pilus biosynthesis protein E* | *tcpE* | P0C6C9 | *OSU_2156* | L1QXB5 | 41 |
| TCP pilus virulence regulatory protein* | *toxT* | Q7BGC0 | *OSU_2153* | L1QW48 | 28 |
| Accessory colonization factor AcfC* | *VC_0841* | Q9KTQ6 | *OSU_2151* | L1QXB0 | 57 |
| Toxin co-regulated pilus biosynthesis protein B† | *tcpB* | P23476 | *OSU_2163* | L1QW56 | 23 |
| Toxin coregulated pilus biosynthesis outer membrane protein C† | *tcpC* | P29481 | *OSU_2161* | L1QXC2 | 27 |
| Toxin coregulated pilus biosynthesis protein Q† | *tcpQ* | P29490 | *OSU_2162* | L1QW23 | 26 |
| Toxin co-regulated pilus biosynthesis protein P, transcriptional activator of ToxT promoter† | *tcpP* | P29485 | *OSU_2154* | L1QWX1 | 28 |
| Toxin co-regulated pilus biosynthesis protein T, putative ATP-binding translocase of TcpA† | *tcpA* | P29480 | *OSU_2157* | L1QW16 | 45 |
| Outer membrane protein U† | *ompU* | P0C6Q6 | *OSU_2985* | L1QTI1 | 69 |
| Maltoporin† | *lamB* | Q56652 | *OSU_1377* | L1QYF6 | 79 |
| Vitamin B12 transporter BtuB† | *btuB* | Q9KVI9 | *btuB* | L1QXI4 | 41 |
| Toxin co-regulated pilus biosynthesis protein E† | *tcpE* | Q7BGC1 | *OSU_2156* | LIQXB5 | 41 |
| MSHA pilin protein MshA† | *VC_0409* | H9L4Q5 | *OSU_1463* | L1QYA8 | 48 |
| Outer membrane protein OmpK† | *VC_2305* | Q9KPR1 | *OSU_0389* | L1R150 | 79 |
| ToxR-activated gene A lipoprotein‡ | *tagA* | P0C6Q7 | *OSU_1601* | L1QYS7 | 42 |
| Toxin coregulated pilin/Toxin coregulated pilin‡ | *tcpA* | Q60153 | *OSU_2164* | L1QWY1 | 35 |

| | | | | | |
|---|---|---|---|---|---|
| TCP pilus virulence regulatory protein‡ | *tcpN* | P0C6D6 | *OSU_2153* | L1QW48 | 28 |
| Toxin coregulated pilus biosynthesis protein B‡ | *tcpB* | P23476 | *OSU_2163* | L1QW56 | 23 |
| Toxin coregulated pilus biosynthesis outer membrane protein C‡ | *tcpC* | P29481 | *OSU_2161* | L1QXC2 | 27 |
| Toxin coregulated pilus biosynthesis protein Q‡ | *tcpQ* | P29490 | *OSU_2162* | L1QW23 | 26 |
| Toxin coregulated pilus biosynthesis protein B‡ | *tcpB* | P23476 | *OSU_2163* | L1QW56 | 23 |
| Toxin coregulated pilus biosynthesis protein P‡ | *tcpP* | P29485 | *OSU_2154* | L1QWX1 | 28 |
| Toxin coregulated pilus biosynthesis protein E‡ | *tcpE* | P0C6C9 | *OSU_2156* | L1QXB5 | 41 |
| Toxin coregulated pilus biosynthesis protein P‡ | *tcpP* | P29485 | *OSU_2154* | L1QWX1 | 28 |
| Toxin coregulated pilus biosynthesis protein T‡ | *tcpT* | P29480 | *OSU_2157* | L1QW16 | 45 |
| Acetyl-coenzyme A carboxyl transferase beta chain* | *accD* | Q9KTA3 | *accD* | L1QUD0 | 99 |
| Acetyl-coenzyme A carboxylase carboxyl transferase subunit alpha* | *accA* | Q9KPW8 | *accA* | L1QU31 | 99 |
| DNA-directed RNA polymerase subunit beta* | *rpoB* | Q9KV30 | *rpoB* | L1R039 | 100 |
| DNA-directed RNA polymerase subunit alpha* | *rpoA* | Q9KP08 | *rpoA* | L1R010 | 100 |
| DNA-directed RNA polymerase subunit beta'* | *rpoC* | Q9KV29 | *OSU_0811* | L1R0N0 | 99 |
| DNA-directed RNA polymerase subunit omega* | *rpoZ* | Q9KNM3 | *rpoZ* | L1QZ53 | 100 |
| Translation initiation factor IF-3* | *infC* | O68844 | *OSU_0528* | L1R0U1 | 99 |
| Translation initiation factor IF-2* | *infB* | Q9KU80 | *infB* | L1QTI8 | 99 |
| Translation initiation factor IF-1* | *infA* | P65128 | *infA* | L1QSS0 | 100 |
| Elongation factor P--(R)-beta-lysine ligase* | *epmA* | Q9KNS6 | *epmA* | L1QZ34 | 99 |
| Elongation factor G 2* | *fusA2* | Q9KPM5 | *fusA* | L1R003 | 99 |
| Elongation factor G 1* | *fusA1* | Q9KUZ7 | *fusA* | L1R1B4 | 99 |
| Elongation factor Tu-A* | *tufA* | Q9KV37 | *tuf* | L1QZU7 | 99 |
| Elongation factor 4* | *lepA* | Q9KPB0 | *lepA* | L1QXD5 | 100 |
| Elongation factor Tu-B* | *tufB* | Q9KUZ6 | *tuf* | L1QZU7 | 100 |
| Elongation factor Ts* | *tsf* | Q9KPV3 | *tsf* | L1QU50 | 100 |
| Magnesium and cobalt efflux protein CorC* | *corC* | Q9KTE3 | *OSU_1839* | L1QXP8 | 100 |

| | | | | | |
|---|---|---|---|---|---|
| Multidrug resistance protein NorM* | *norM* | Q9KRU4 | *OSU_0958* | L1R0S0 | 99 |
| DNA gyrase subunit B* | *gyrB* | Q9KVX3 | *gyrB* | L1R0B1 | 99 |
| DNA gyrase subunit A* | *gyrA* | Q9KSJ8 | *gyrA* | L1R046 | 99 |
| DNA topoisomerase 1* | *topA* | Q9KRB2 | *OSU_3325* | L1QSW2 | 99 |
| Quinolinate synthase A* | *nadA* | Q9KR14 | *nadA* | L1QYF4 | 99 |
| Hemagglutinin/proteinase* | *hap* | P24153 | *OSU_2585* | L1QUI8 | 99 |
| Thymidylate synthase* | *thyA* | O66108 | *thyA* | L1QTZ3 | 99 |
| Conserved uncharacterized protein CreA* | *CreA* | Q9KLE6 | *OSU_0610* | L1R1B8 | 100 |
| Methyl-accepting chemotaxis protein, hemolysin secretion protein HylB* | *hlyB* | P15492 | *OSU_0767* | L1R059 | 99 |
| Elongation factor Tu* | *tufA* | Q9KV37 | *tuf* | L1QZU7 | 99 |
| Peptide chain release factor 3* | *prfC* | Q9KU64 | *prfC* | L1QTP4 | 100 |
| Peptide chain release factor 1* | *prfA* | Q9KQ25 | *prfA* | L1QTF3 | 100 |
| Transcription elongation factor GreA* | *greA* | Q9KU89 | *greA* | L1QTM2 | 100 |
| Elongation factor P* | *efp* | Q9KNS1 | *efp* | L1QZU9 | 100 |
| Thiol:disulfide interchange protein DsbA* | *dsbA* | P32557 | *OSU_0224* | L1R1I7 | 99 |
| Protein Hfq* | *hfq* | Q9KV11 | *hfq* | L1QZW2 | 100 |
| CAI-1 autoinducer synthase* | *cqsA* | Q9KM65 | *OSU_3417* | L1QTS5 | 86 |
| $Na^{+}$-translocating NADH-quinone reductase subunit A* | *nqrA* | Q9KPS1 | *nqrA* | L1R115 | 99 |
| $Na^{+}$-translocating NADH-quinone reductase subunit B* | *nqrB* | Q9KPS2 | *nqrB* | L1R160 | 100 |
| $Na^{+}$-translocating NADH-quinone reductase subunit D* | *nqrD* | Q9X4Q6 | *nqrD* | L1R1A8 | 100 |
| $Na^{+}$-translocating NADH-quinone reductase subunit E* | *nqrE* | Q9X4Q7 | *nqrE* | L1R2D7 | 100 |
| $Na^{+}$-translocating NADH-quinone reductase subunit F* | *nqrF* | Q9X4Q8 | *nqrF* | L1R118 | 99 |
| Regulatory protein LuxO* | *luxO* | Q9KT84 | *OSU_0967* | L1QZS0 | 100 |
| Phosphorelay protein LuxU* | *luxU* | Q9KT83 | *OSU_0968* | L1R0S8 | 100 |
| Cholera toxin transcriptional activator* | *toxR* | P15795 | *OSU_2698* | L1QUT2 | 99 |
| CAI-1 autoinducer sensor kinase/phosphatase CqsS* | *cqsS* | Q9KM66 | *OSU_3416* | L1QSS7 | 92 |
| Chaperone protein DnaJ* | *dnaJ* | O34242 | *dnaJ* | L1R0S1 | 100 |

| | | | | | |
|---|---|---|---|---|---|
| Iron-regulated outer membrane virulence protein* | *irgA* | P0C6R0 | *OSU_2864* | L1QVJ2 | 99 |
| Iron-regulated virulence regulatory protein IrgB* | *irgB* | P0C6D1 | *OSU_2865* | L1QU69 | 100 |
| Transcriptional activator HlyU* | *hlyU* | P52695 | *OSU_3031* | L1QTR2 | 100 |
| Chaperone protein DnaK* | *dnaK* | O34241 | *dnaK* | L1R081 | 100 |
| Transcriptional activator protein NhaR* | *nhaR* | P52692 | *OSU_3030* | L1QTL7 | 100 |
| Outer membrane protein OmpV* | *ompV* | P06111 | *OSU_0584* | L1R0I3 | 99 |
| Protein GrpE* | *grpE* | O30862 | *grpE* | L1QZY3 | 99 |
| Chaperone protein HtpG* | *htpG* | P22359 | *htpG* | L1QVT6 | 99 |
| RNA polymerase sigma factor RpoS* | *rpoS* | O51804 | *OSU_2811* | L1QU58 | 99 |
| Amidophosphoribosyltransferase* | *VC_1004* | Q9KT99 | *OSU_2719* | L1QVR0 | 99 |
| Bacteriocin production protein* | *VC_1003* | Q9KTA0 | *OSU_2718* | L1QUQ6 | 100 |
| DedD protein* | *VC_1002* | Q9KTA1 | *OSU_2717* | L1QV96 | 99 |
| Elongation factor P-like protein* | *VC_1209* | Q9KSP7 | *OSU_0847* | L1R107 | 100 |
| Sui1 family protein* | *VC_A0570* | Q9KM19 | *OSU_0889* | L1R0U9 | 100 |
| Transcriptional regulator, MerR family* | *VC0277* | Q9KV79 | *OSU_0510* | L1QXC6 | 100 |
| Transcriptional regulator, MerR family* | *VC_A0056* | Q9KMQ6 | *OSU_0510* | L1R1N3 | 99 |
| Transcriptional regulator, MerR family, associated with photolyase* | *VC_A0056* | Q9KNA9 | *OSU_1511* | L1QYH0 | 99 |
| Topoisomerase IV subunit A* | *VC_2430* | Q9KPE0 | *OSU_2016* | L1QWQ7 | 99 |
| Topoisomerase IV subunit B* | *VC_2431* | Q9KPD9 | *OSU_2017* | L1QXP9 | 99 |
| Chromate transport protein ChrA* | *VC_2339* | Q9KPM8 | *OSU_0369* | L1R1F3 | 95 |
| Dihydrofolate synthase* | *VC_1001* | Q9KTA2 | *OSU_2716* | L1QUG8 | 99 |
| Transcriptional regulator, LysR family* | *VC_1049* | Q9KT56 | *OSU_2393* | L1QWG4 | 100 |
| Hcp protein* | *VC_1415, VC_A0017* | H9L4Q3 | *OSU_2925* | L1QU26 | 100 |
| Putative uncharacterized protein* | *VC_A0112* | Q9KN53 | *OSU_1569* | L1QXT8 | 99 |
| IcmF-related protein* | *VC_A0120* | Q9KN45 | *OSU_1577* | L1QY50 | 99 |
| ClpB protein* | *VC_A0116* | Q9KN49 | *OSU_1573* | L1QZ48 | 99 |

| | | | | | |
|---|---|---|---|---|---|
| Sigma-54 dependent transcriptional regulator* | *VC_A0117* | Q9KN48 | *OSU_1574* | L1QXU1 | 99 |
| Putative uncharacterized protein* | *VC_A0121* | Q9KN44 | *OSU_1578* | L1QZ51 | 99 |
| Putative uncharacterized protein* | *VC_A0110* | Q9KN55 | *OSU_1567* | L1QY39 | 100 |
| Putative uncharacterized protein* | *VC_A0111* | Q9KN54 | *OSU_1568* | L1QZ45 | 99 |
| Putative uncharacterized protein* | *VC_A0114* | Q9KN51 | *OSU_1571* | L1QYQ3 | 100 |
| Putative uncharacterized protein* | *VC_A0113* | Q9KN52 | *OSU_1570* | L1QXY9 | 100 |
| Putative uncharacterized protein* | *VC_A0119* | Q9KN46 | *OSU_1576* | L1QYQ6 | 99 |
| Putative uncharacterized protein* | *VC_A0115* | Q9KN50 | *OSU_1572* | L1QY45 | 99 |
| Aldehyde dehydrogenase* | *VC1819* | Q9KR28 | *OSU_1623* | L1QYU8 | 99 |
| Type 4 prepilin-like proteins leader peptide-processing enzyme* | *VC_2426* | H9L4S3 | *OSU_2012* | L1QXP5 | 99 |
| Oligopeptide transport ATP-binding protein OppD† | *oppD* | Q9KT11 | *OSU_0556* | L1R0Q6 | 99 |
| Magnesium and cobalt efflux protein CorC† | *corC* | Q9KTE3 | *OSU_1839* | L1QXP8 | 100 |
| 3-dehydroquinate synthase† | *aroB* | Q9KNV2 | *aroB* | L1QZR9 | 99 |
| Type IV fimbrial assembly protein PilC† | *pilC* | Q9X4G9 | *OSU_2011* | L1QWQ2 | 100 |
| Type I secretion outer membrane protein, TolC† | *tolC* | Q9K2Y1 | *OSU_2022* | L1QXQ3 | 99 |
| Dephospho-CoA kinase (EC 2.7.1.24)† | *coaE* | Q9KPE3 | *coaE* | L1QWE4 | 99 |
| Type II secretion system protein G† | *epsG* | P45773 | *OSU_0024* | L1R303 | 100 |
| Type II secretion system protein C† | *epsC* | P45777 | *OSU_0020* | L1R2E1 | 99 |
| Type II secretion system protein H† | *epsH* | P45774 | *OSU_0025* | L1R2E6 | 99 |
| Type II secretion system protein L† | *epsL* | P45782 | *OSU_0029* | L1R308 | 99 |
| Type II secretion system protein E† | *epsE* | P37093 | *OSU_0022* | L1R249 | 100 |
| Type II secretion system protein F† | *epsF* | P45780 | *OSU_0023* | L1R290 | 99 |
| Type II secretion system protein M† | *epsM* | P41851 | *OSU_0030* | L1R2F1 | 99 |
| Type II secretion system protein I† | *epsI* | P45775 | *OSU_0026* | L1R3H8 | 100 |
| Type II secretion system protein J† | *epsJ* | P45776 | *OSU_0027* | L1R254 | 99 |
| Type II secretion system protein D† | *epsD* | P45779 | *OSU_0021* | L1R3H4 | 97 |
| Type II secretion system protein K† | *epsK* | P45781 | *OSU_0028* | L1R295 | 100 |

| | | | | | |
|---|---|---|---|---|---|
| Type II secretion system protein N† | *epsN* | P45784 | *OSU_0031* | L1R3I2 | 99 |
| ClpB protein† | *clpB* | Q9KU18 | *OSU_3062* | L1QUM8 | 99 |
| Signal recognition particle receptor protein FtsY† | *ftsY* | Q9KVJ6 | *ftsY* | L1QZB6 | 99 |
| Signal recognition particle, subunit Ffh† | *ffh* | Q9KUG1 | *ffh* | L1QXG4 | 99 |
| Sec-independent protein translocase protein TatA† | *tatA* | P57051 | *tatA* | L1QWT4 | 100 |
| Sec-independent protein translocase protein TatB† | *tatB* | P57063 | *tatB* | L1QW18 | 100 |
| Sec-independent protein translocase protein TatC† | *tatC* | Q9KVQ3 | *tatC* | L1QVX9 | 99 |
| H(+)/Cl(-) exchange transporter ClcA† | *clcA* | Q9KM62 | *clcA* | L1QSI8 | 99 |
| Transcriptional activator HlyU† | *hlyU* | P52695 | *OSU_3031* | L1QTR2 | 100 |
| Transcriptional activator NhaR† | *nhaR* | P52692 | *OSU_3030* | L1QTL7 | 100 |
| Biopolymer transport protein exbD1† | *exbD1* | O52044 | *OSU_2641* | L1QUT3 | 100 |
| Biopolymer transport protein exbD2† | *exbD2* | Q9ZHV9 | *OSU_3210* | L1QT77 | 100 |
| Protein TonB† | *tonB* | O52042 | *OSU_2639* | L1QW13 | 99 |
| Protein-L-isoaspartate O-methyltransferase (EC 2.1.1.77)† | *pcm* | Q9KUI8 | *pcm* | L1QUD6 | 100 |
| Protein TolB† | *tolB* | Q9KR11 | *tolB* | L1QYQ7 | 100 |
| Lipid A export ATP-binding/permease protein MsbA† | *msbA* | Q9KQW9 | *OSU_1317* | L1QZV3 | 99 |
| $Na^+$-translocating NADH-quinone reductase subunit B† | *nqrB* | Q9KPS2 | *nqrB* | L1R160 | 100 |
| $Na^+$-translocating NADH-quinone reductase subunit E† | *nqrE* | Q9X4Q7 | *nqrE* | L1R2D7 | 100 |
| $Na^+$-translocating NADH-quinone reductase subunit D† | *nqrD* | Q9X4Q6 | *nqrD* | L1R1A8 | 100 |
| $Na^+$-translocating NADH-quinone reductase subunit A† | *nqrA* | Q9KPS1 | *nqrA* | L1R115 | 99 |
| Protein translocase subunit SecY† | *secY* | P78283 | *secY* | L1QZ07 | 100 |
| PTS system mannitol-specific EIICBA component† | *mtlA* | Q9KKQ7 | *OSU_1391* | L1QZ16 | 99 |
| Biopolymer transport protein exbB1† | *exbB1* | O52043 | *OSU_2640* | L1QUP7 | 99 |
| Electron transport complex protein RnfA† | *rnfA* | Q9KT86 | *rnfA* | L1QVB2 | 100 |
| Electron transport complex protein RnfD† | *rnfD* | Q9KT89 | *rnfD* | L1QVR9 | 99 |
| Maltose transport system permease protein MalF† | *malF* | Q9KL06 | *OSU_2670* | L1QUS8 | 99 |
| Maltose transport system permease protein MalG† | *malG* | Q9KL07 | *OSU_2669* | L1QW44 | 100 |

| | | | | | |
|---|---|---|---|---|---|
| Electron transport complex protein RnfE† | *rnfE* | Q9KT91 | *rnfE* | L1QVA6 | 99 |
| Electron transport complex protein RnfG† | *rnfG* | Q9KT90 | *rnfG* | L1QUR6 | 100 |
| Outer-membrane lipoprotein carrier protein† | *lolA* | P57069 | *lolA* | L1QW77 | 100 |
| Membrane protein insertase YidC† | *yidC* | Q9KVY4 | *yidC* | L1R0A1 | 99 |
| Electron transport complex protein RnfB† | *rnfB* | Q9KT87 | *rnfB* | L1QUI5 | 100 |
| Probable D-methionine transport system permease protein MetI† | *metI* | Q9KTJ6 | *OSU_2275* | L1QVS7 | 100 |
| Outer-membrane lipoprotein LolB† | *lolB* | P57070 | *lolB* | L1QT50 | 99 |
| $Na^{+}/H^{+}$ antiporter NhaB† | *nhaB* | Q9KQU7 | *nhaB* | L1QSX1 | 100 |
| Probable D-methionine-binding lipoprotein MetQ† | *metQ* | Q9KTJ7 | *OSU_2276* | L1QWJ2 | 100 |
| L-alanine exporter AlaE† | *alaE* | Q9KR19 | *alaE* | L1QZ89 | 99 |
| Hemolysin secretion protein† | *hlyB* | P15492 | *OSU_0767* | L1R059 | 99 |
| *Vibrio* bactin receptor† | *viuA* | P0C6R1 | *OSU_2074* | L1QX43 | 99 |
| $Fe^{3+}$ ions import ATP-binding protein FbpC† | *fbpC* | Q9KLQ5 | *OSU_0268* | L1R1I6 | 99 |
| Serine/threonine transporter SstT† | *sstT* | Q9KNC9 | *sstT* | L1QWQ1 | 99 |
| Probable oxaloacetate decarboxylase beta chain† | *oadB* | Q9KTU5 | *OSU_0160* | L1R1W6 | 100 |
| Probable oxaloacetate decarboxylase gamma chain 1† | *oadG1* | Q9KUH2 | *oadG* | L1QXH3 | 99 |
| Cell volume regulation protein A homolog† | *cvrA* | Q9KNM9 | *OSU_1254* | L1QZ33 | 99 |
| Protein CysZ homolog† | *cysZ* | Q9KTD3 | *cysZ* | L1QWZ9 | 99 |
| Disulfide bond formation protein B† | *dsbB* | Q9KQU6 | *dsbB* | L1QTX6 | 99 |
| Sulfate/thiosulfate import ATP-binding protein CysA† | *cysA* | Q9KUI0 | *OSU_2805* | L1QU09 | 99 |
| Methionine import ATP-binding protein MetN† | *metN* | Q9KTJ5 | *OSU_2274* | L1QVP3 | 99 |
| Spermidine/putrescine import ATP-binding protein PotA† | *potA* | Q9KS33 | *OSU_0057* | L1R3E2 | 99 |
| sn-glycerol-3-phosphate import ATP-binding protein UgpC† | *ugpC* | Q9KRT4 | *OSU_3203* | L1QUG5 | 92 |
| Zinc import ATP-binding protein ZnuC† | *znuC* | Q9KQB8 | *OSU_0319* | L1R1L5 | 100 |
| Vitamin B12 import system permease protein BtuC† | *btuC* | Q9KSL2 | *btuC* | L1R090 | 100 |

| | | | | | |
|---|---|---|---|---|---|
| Large-conductance mechanosensitive channel† | *mscL* | Q9KLX9 | *mscL* | L1R281 | 99 |
| Probable anaerobic C4-dicarboxylate transporter DcuC† | *dcuC* | Q9KLS6 | *OSU_0252* | L1R2U7 | 100 |
| Protein translocase subunit SecE† | *secE* | Q9KV36 | *OSU_0818* | L1R163 | 100 |
| Hemin import ATP-binding protein HmuV† | *hmuV* | Q9KL34 | *OSU_2644* | L1QW17 | 100 |
| Galactose/methyl galactoside import ATP-binding protein MglA† | *mglA* | Q9KSD1 | *OSU_0591* | L1R0M8 | 99 |
| Phosphate import ATP-binding protein PstB 1† | *pstB1* | Q9KU04 | *OSU_3497* | L1QSM9 | 100 |
| Phosphate import ATP-binding protein PstB 2† | *pstB2* | Q9KN92 | *OSU_1527* | L1QXX2 | 100 |
| Ribose import ATP-binding protein RbsA† | *rbsA* | Q9KN37 | *OSU_1583* | L1QZ55 | 100 |
| LPS-assembly protein LptD† | *lptD* | Q9KUR9 | *lptD* | L1QY85 | 99 |
| Cytochrome c biogenesis ATP-binding export protein CcmA† | *ccmA* | Q9KQE3 | *OSU_0995* | L1QZI4 | 100 |
| Molybdenum import ATP-binding protein ModC† | *modC* | Q9KLL9 | *OSU_1636* | L1QXF8 | 99 |
| Lipoprotein-releasing system ATP-binding protein LolD† | *lolD* | P57066 | *OSU_1321* | L1QYV4 | 100 |
| Phosphocarrier protein HPr† | *ptsH* | Q9KTD6 | *OSU_1846* | L1QY49 | 100 |
| ATP synthase subunit a† | *atpB* | Q9KNG9 | *atpB* | L1R136 | 100 |
| ATP synthase subunit b† | *atpF* | Q9KNH1 | *atpF* | L1R088 | 99 |
| ATP synthase subunit alp† | *atpA* | Q9KNH3 | *atpA* | L1R0H5 | 100 |
| ATP synthase subunit beta† | *atpD* | Q9KNH5 | *atpD* | L1R0D8 | 100 |
| ATP synthase subunit delta† | *atpH* | Q9KNH2 | *atpH* | L1R1K7 | 100 |
| ATP synthase epsilon chain† | *atpC* | Q9KNH6 | *atpC* | L1R084 | 100 |
| ATP synthase gamma chain† | *atpG* | Q9KNH4 | *atpG* | L1R130 | 100 |
| PTS system N-acetylmuramic acid-specific EIIBC component† | *murP* | Q9KVD9 | *OSU_3243* | L1QT65 | 100 |
| $Na^{+}$-translocating NADH-quinone reductase subunit C† | *nqrC* | P0C6E0 | *nqrC* | L1R1W9 | 100 |
| Thiol:disulfide interchange protein DsbD† | *dsbD* | Q9KNN1 | *dsbD* | L1QZC7 | 99 |
| Protein translocase subunit SecA† | *secA* | Q9KPH4 | *secA* | L1R1X0 | 99 |

| | | | | | |
|---|---|---|---|---|---|
| Maltose/maltodextrin import ATP-binding protein MalK† | *malK* | Q9KL04 | *OSU_2672* | L1QVR3 | 99 |
| Periplasmic nitrate reductase† | *napA* | Q9KLR4 | *napA* | L1R1H7 | 99 |
| ATP synthase subunit c† | *atpE* | Q9KNH0 | *OSU_0661* | L1R0E2 | 100 |
| $Na^+/H^+$ antiporter NhaA† | *nhaA* | O85187 | *nhaA* | L1R0L7 | 99 |
| Protein-export membrane protein SecF† | *secF* | Q9KTY6 | *secF* | L1R3A0 | 100 |
| Protein translocase subunit SecD† | *secD* | Q9KLQ0 | *secD* | L1QT46 | 99 |
| Protein translocase subunit SecD† | *secD* | Q9KTY7 | *secD* | L1R1X7 | 99 |
| Sec-independent protein translocase protein TatA HAMAP-Rule† | *tatA* | Q9KM55 | *tatA* | L1QST7 | 93 |
| Lipid II flippase FtsW† | *ftsW* | Q9KPG6 | *ftsW* | L1R1U2 | 100 |
| Oligopeptide ABC transporter, permease protein† | *VC_1093* | Q9KT12 | *OSU_2437* | L1QVM1 | 100 |
| ABC-type tungstate transport system, permease protein† | *VC_1523* | Q9KRW0 | *OSU_0933* | L1QZY7 | 100 |
| Peptide transport system ATP-binding protein SapF† | *VC_1684* | Q9KRF8 | *OSU_1952* | L1QWR8 | 99 |
| MSHA biogenesis protein MshE† | *VC_0405* | Q9KUV7 | *OSU_1459* | L1QZ98 | 99 |
| MSHA biogenesis protein MshF† | *VC_0407* | Q9KUV5 | *OSU_1461* | L1QY48 | 94 |
| MSHA biogenesis protein MshI† | *VC_0399* | Q9KUW0 | *OSU_1453* | L1QYA0 | 99 |
| MSHA biogenesis protein MshJ† | *VC_0400* | H9L4P3 | *OSU_1454* | L1QZ95 | 100 |
| MSHA biogenesis protein MshK† | *VC_0401* | H9L4T2 | *OSU_1455* | L1QXZ0 | 99 |
| MSHA biogenesis protein MshL† | *VC_0402* | Q9KUV9 | *OSU_1456* | L1QY43 | 99 |
| MSHA biogenesis protein MshM† | *VC_0403* | H9L4Q2 | *OSU_1457* | L1QYV6 | 100 |
| MSHA biogenesis protein MshN† | *VC_0404* | Q9KUV8 | *OSU_1458* | L1QYA4 | 99 |
| MSHA pilin protein MshB† | *VC_0408* | Q9KUV4 | *OSU_1462* | L1QYW0 | 99 |
| MSHA pilin protein MshC† | *VC_0410* | Q9KUV3 | *OSU_1464* | L1QZA3 | 96 |
| MSHA pilin protein MshD† | *VC_0411* | Q9KUV2 | *OSU_1465* | L1QXZ7 | 97 |
| Type 4 prepilin-like proteins leader peptide-processing enzyme† | *VC_2426* | H9L4S3 | *OSU_2012* | L1QXP5 | 99 |
| Twitching motility protein PilT† | *VC_0463* | Q9KUQ2 | *OSU_1900* | L1QWX4 | 99 |

| | | | | | |
|---|---|---|---|---|---|
| Twitching motility protein PilT† | *VC_0462* | Q9KUQ3 | *OSU_1901* | L1QXN7 | 100 |
| Type IV fimbrial assembly, ATPase PilB† | *VC_2424* | Q9KPE4 | *OSU_2010* | L1QXA2 | 99 |
| Type IV pilus biogenesis protein PilP† | *VC_2631* | Q9KNU9 | *OSU_1184* | L1QYW2 | 100 |
| Microcin H47 secretion protein† | *VC_1447* | H9L4R8 | *OSU_0075* | L1R2Y2 | 100 |
| RTX toxins determinant A† | *VC_1451* | Q9KS12 | *OSU_0079* | L1R275 | 96 |
| Toxin secretion transporter, putative† | *VC_1446* | Q9KS15 | *OSU_0074* | L1R271 | 99 |
| Mutator MutT protein† | *VC_2392* | Q9KPH6 | *OSU_0125* | L1R1S9 | 100 |
| Flagellum-specific ATP synthase FliI† | *VC_2130* | Q9KQ71 | *OSU_2497* | L1QV16 | 99 |
| General secretion pathway protein A† | *VC_2445* | Q9KPC7 | *OSU_2032* | L1QXR1 | 99 |
| General secretion pathway protein B† | *VC_2444* | Q9KPC8 | *OSU_2031* | L1QWS0 | 99 |
| IcmF-related protein† | *VC_A0120* | Q9KN45 | *OSU_1577* | L1QY50 | 99 |
| Sigma-54 dependent transcriptional regulator† | *VC_A0117* | Q9KN48 | *OSU_1574* | L1QXU1 | 99 |
| Uncharacterized protein ImpA† | *VC_A0119* | Q9KN46 | *OSU_1576* | L1QYQ6 | 99 |
| TRAP-type C4-dicarboxylate transport system, large permease component† | *VC_1927* | Q9KQS1 | *OSU_1749* | L1QUN1 | 100 |
| $Na^{+/}$ $H^+$ antiporter subunit C† | *VC_A0156* | Q9KN10 | *OSU_1608* | L1QZ76 | 100 |
| NptA protein/ Sodium-dependent phosphate transporter† | *VC_0676* | H9L4N7 | *OSU_3029* | L1QUZ6 | 100 |
| Phosphoglycerate transport regulatory protein PgtC, putative† | *VC_A0706* | Q9KLN7 | *OSU_1652* | L1QXM1 | 99 |
| Phosphoglycerate transport system sensor protein PgtB (EC 2.7.3.-)† | *VC_A0705* | Q9KLN8 | *OSU_1653* | L1QYD4 | 99 |
| Phosphoglycerate transport system transcriptional regulatory protein PgtA† | *VC_A0704* | Q9KLN9 | *OSU_1654* | L1QXS4 | 100 |
| TolA protein† | *VC_1837* | Q9KR10 | *OSU_1277* | L1QZR3 | 100 |
| TonB-dependent receptor† | *VC_A0064* | Q9KNA1 | *OSU_1519* | L1QXL6 | 99 |
| Tricarboxylate transport membrane protein TctA† | *VC_1332* | Q9KSC6 | *OSU_2371* | L1QWI4 | 99 |
| Tricarboxylate transport protein TctB† | *VC_1333* | Q9KSC5 | *OSU_2370* | L1QVR8 | 100 |
| Tricarboxylate transport protein TctC† | *VC_1334* | Q9KSC4 | *OSU_2369* | L1QVN4 | 100 |

| | | | | | |
|---|---|---|---|---|---|
| Putative transport protein VC_1145† | *VC_1145* | Q9KSW1 | *OSU_2770* | L1QV84 | 100 |
| Uncharacterized transporter VC_0195† | *VC_0195* | Q9KVF1 | *OSU_3237* | L1QTQ2 | 99 |
| C4-dicarboxylate transport sensor protein† | *VC_1925* | Q9KQS3 | *OSU_3075* | L1QU74 | 99 |
| C4-dicarboxylate transport sensor protein, putative† | *VC_A0141* | Q9KN25 | *OSU_1595* | L1QY08 | 99 |
| NupC family protein† | *VC_2352* | Q9KPL5 | *OSU_0089* | L1R1K2 | 100 |
| Histidine protein kinase PhoR† | *VC_0720* | Q9KU10 | *OSU_3491* | L1QT76 | 99 |
| Phosphoenolpyruvate-protein phosphotransferase† | *VC_0965* | Q9KTD7 | *OSU_1845* | L1QX64 | 99 |
| Tyrosine-specific transport protein† | *VC_A0772* | Q9KLH2 | *OSU_0631* | L1R0R7 | 100 |
| Ferrous iron transport protein B† | *VC_2077* | Q9KQC3 | *OSU_0324* | L1R1L9 | 99 |
| Heme transport protein HutA† | *VC_A0576* | Q9KM13 | *OSU_0883* | L1QZU0 | 99 |
| Tryptophan-specific transport protein† | *VC_A0160* | Q9KN06 | *OSU_1612* | L1QY80 | 99 |
| Cation transport ATPase, E1-E2 family† | *VC_1033* | Q9KT72 | *OSU_0977* | L1QZT0 | 99 |
| Cation transport ATPase, E1-E2 family† | *VC_1437* | Q9KS24 | *OSU_0066* | L1R2B1 | 99 |
| Trk system potassium uptake protein† | *VC_2756* | Q9KNI3 | *OSU_0648* | L1R0G7 | 99 |
| PTS system, fructose-specific IIBC component† | *VC_A0516* | Q9KM72 | *OSU_3410* | L1QTC0 | 99 |
| Thiamine ABC transporter, permease protein, putative† | *VC_2538* | Q9KP41 | *OSU_1093* | L1QZY0 | 99 |
| Glutathione-regulated potassium-efflux system protein† | *VC_2606* | Q9KNX4 | *OSU_1158* | L1R045 | 99 |
| Amino acid ABC transporter, permease protein† | *VC_1361* | Q9KS98 | *OSU_2344* | L1QVK8 | 99 |
| ABC transporter, permease protein† | *VC_1524* | Q9KRW0 | *OSU_0933* | L1QZY7 | 100 |
| Peptide ABC transporter, permease protein, putative† | *VC_A0589* | Q9KM00 | *OSU_0555* | L1R0L8 | 99 |
| Amino acid ABC transporter, permease protein† | *VC_A1038* | Q9KKR4 | *OSU_1387* | L1QYG5 | 99 |
| Phosphate ABC transporter, permease protein† | *VC_0724* | Q9KU06 | *OSU_3495* | L1QSD1 | 99 |
| Arginine ABC transporter, permease protein† | *VC_A0757* | Q9KLI7 | *OSU_1068* | L1R025 | 100 |
| Amino acid ABC transporter, permease protein† | *VC_1861* | Q9KQY6 | *OSU_1299* | L1QYM9 | 100 |
| Putative uncharacterized protein† | *VC_1211* | Q9KSP5 | *OSU_0845* | L1R0I7 | 99 |
| Amino acid ABC transproter, permease protein† | *VC_0009* | Q9KVX9 | *OSU_0674* | L1R1M6 | 100 |
| Amino acid ABC transporter, permease protein† | *VC_1862* | Q9KQY5 | *OSU_1300* | L1QZB9 | 100 |
| Iron(III) ABC transporter, permease protein† | *VC_0609* | Q9KUB4 | *OSU_3333* | L1QSR7 | 99 |

| | | | | | |
|---|---|---|---|---|---|
| Molybdenum ABC transporter, permease protein† | *VC_A0725* | Q9KLL8 | *OSU_1635* | L1QYR3 | 99 |
| Peptide ABC transporter, permease protein† | *VC_0173* | Q9KVH3 | *OSU_3228* | L1QT49 | 100 |
| ABC transporter, permease protein† | *VC_A1100* | Q9KKK4 | *OSU_2951* | L1QV48 | 99 |
| Iron(III) ABC transporter, permease protein† | *VC_A0686* | Q9KLQ6 | *OSU_0267* | L1R2W0 | 99 |
| Amino acid ABC transporter, permease protein / Glutamate Aspartate transport system permease protein GltK† | *VC_1360* | Q9KS99 | *OSU_2345* | L1QVP8 | 99 |
| Peptide ABC transporter, permease protein† | *VC_1682* | Q9KRG0 | *OSU_1954* | L1QX00 | 100 |
| Arginine ABC transporter, permease protein† | *VC_A0758* | Q9KLI6 | *OSU_1069* | L1QZG8 | 99 |
| ABC transporter, permease protein† | *VC_A0601* | Q9KLZ0 | *OSU_0460* | L1R142 | 99 |
| Phosphate ABC transporter, permease protein† | *VC_A0072* | Q9KN93 | *OSU_1526* | L1QYI6 | 99 |
| Phosphate ABC transporter, permease protein† | *VC_0725* | Q9KU05 | *OSU_3496* | L1QT80 | 99 |
| Putative uncharacterized protein† | *VC_0689* | Q9KU40 | *OSU_3041* | L1QTR9 | 99 |
| Lipopolysaccharide export system protein LptC† | *VC_2525* | Q9KP51 | *OSU_1081* | L1QZF0 | 100 |
| Glutathione-regulated potassium-efflux system protein KefB, putative† | *VC_0992* | Q9KTB1 | *OSU_2706* | L1QUL2 | 100 |
| Transporter, BCCT family / High-affinity choline uptake protein BetT† | *VC_1279* | Q9KSH7 | *OSU_0744* | L1R0C4 | 99 |
| Heme exporter protein B† | *VC_2056* | Q9KQE4 | *OSU_0996* | L1R092 | 99 |
| Sulfate ABC transporter, permease protein† | *VC_0540* | Q9KUI1 | *OSU_2806* | L1QU52 | 100 |
| TonB receptor-related protein† | *VC_A0625* | Q9KLW6 | *OSU_1888* | L1QY15 | 99 |
| Sulfate ABC transporter, permease protein† | *VC_0539* | Q9KUI2 | *OSU_2807* | L1QUX0 | 99 |
| PTS system, glucose-specific IIA component† | *VC_0964* | Q9KTD8 | *OSU_1844* | L1QXQ2 | 100 |
| Proton/peptide symporter family protein/Di/tripeptide permease DtpA† | *VC_0988* | Q9KTB5 | *OSU_2702* | L1QVD6 | 99 |
| MotA/TolQ/ExbB proton channel family protein† | *VC_1839* | Q9KR08 | *OSU_1279* | L1QYL0 | 99 |
| Uracil permease† | *VC_2171* | Q9KQ33 | *OSU_2532* | L1QV47 | 99 |
| Multidrug transporter, putative† | *VC_1597* | Q9KRN9 | *OSU_1052* | L1QZL2 | 99 |

| | | | | | |
|---|---|---|---|---|---|
| Regulatory protein UhpC, putative/ Phosphoglycerate transporter protein PgtP† | *VC_A0707* | Q9KLN6 | *OSU_1651* | L1QXH4 | 99 |
| Transporter, NadC family/ Di-and tricarboxylate transporter† | *VC_A0025* | Q9KNE0 | *OSU_1928* | L1QY03 | 99 |
| Multidrug resistance transporter, Bcr/CflA family† | *VC_0069* | Q9KVS0 | *OSU_2210* | L1QWA2 | 100 |
| PTS system, fructose-specific IIBC component† | *VC_1821* | Q9KR26 | *OSU_1263* | L1QYE4 | 99 |
| Putative uncharacterized protein† | *VC_A0972* | Q9KKX8 | *OSU_1776* | L1QXD4 | 99 |
| Thiosulfate ABC transporter, periplasmic thiosulfate-binding protein / Sulfate and thiosulfate binding protein CysP† | *VC_0538* | Q9KUI3 | *OSU_2808* | L1QUD2 | 99 |
| Regulatory protein UhpC / Hexose phosphate uptake regulatory protein UhpC† | *VC_A0684* | Q9KLQ8 | *OSU_0265* | L1R2B7 | 99 |
| PTS system, fructose-specific IIB component† | *VC_1823* | Q9KR24 | *OSU_1265* | L1QZ87 | 100 |
| TonB2 protein/Ferric siderophore transport system, periplasmic binding protein TonB† | *VC_1544* | Q9KRU0 | *OSU_3211* | L1QU12 | 100 |
| Transporter† | *VC_2283* | Q9KPS9 | *OSU_0409* | L1R170 | 99 |
| Sodium/proline symporter† | *VC_A1071* | Q9KKN1 | *OSU_2979* | L1QUS1 | 99 |
| NADH dehydrogenase, putative/ $Na^+/H^+$ antiporter subunit A / $Na^+/H^+$ antiporter subunit B† | *VC_A0157* | Q9KN09 | *OSU_1609* | L1QXW8 | 99 |
| NADH dehydrogenase, putative/ $Na^+/H^+$ antiporter subunit D† | *VC_A0155* | Q9KN11 | *OSU_1607* | L1QY77 | 99 |
| Putative uncharacterized protein† | *VC_1261* | Q9KSJ5 | *OSU_0726* | L1R0E6 | 99 |
| C4-dicarboxylate-binding periplasmic protein† | *VC_1929* | Q9KQR9 | *OSU_3079* | L1QTD3 | 100 |
| Peptide ABC transporter, ATP-binding protein/(GlcNAc)2 ABC transporter, ATP-binding component 1† | *VC_0617* | Q9KUA6 | *OSU_3341* | L1QU17 | 99 |
| Sulfate permease family protein† | *VC_A0077* | Q9KN88 | *OSU_1532* | L1QXX9 | 99 |
| Oligopeptide ABC transporter, ATP-binding protein† | *VC_1094* | Q9KT11 | *OSU_2438* | L1QWK5 | 99 |

| | | | | | |
|---|---|---|---|---|---|
| Peptide ABC transporter, ATP-binding protein† | *VC_0616* | Q9KUA7 | *OSU_3340* | L1QT04 | 99 |
| Multidrug resistance protein† | *VC_2761* | Q9KNH8 | *OSU_0653* | L1R0H2 | 99 |
| Anaerobic glycerol-3-phosphate dehydrogenase, subunit C† | *VC_A0749* | Q9KLJ5 | *OSU_2887* | L1QTZ4 | 99 |
| Multidrug resistance protein, putative† | *VC_0914* | Q9KTI8 | *OSU_1335* | L1QYH5 | 99 |
| Sulfate permease family protein† | *VC_2031* | Q9KQG7 | *OSU_1697* | L1QXN8 | 99 |
| Formate dehydrogenase, cytochrome B556 subunit† | *VC_1511* | Q9KRX2 | *OSU_0920* | L1QZL6 | 100 |
| NupC family protein† | *VC_A0179* | Q9KMY7 | *OSU_3478* | L1QSG2 | 100 |
| Putative uncharacterized protein/1-acyl-sn-glycerol-3-phosphate acyltransferase† | *VC_1966* | Q9KQN2 | *OSU_3111* | L1QTQ8 | 99 |
| Multidrug resistance protein† | *VC_1634* | Q9KRK6 | *OSU_0873* | L1QZR8 | 99 |
| Putative uncharacterized protein† | *VC_0480* | Q9KUN5 | *OSU_2860* | L1QU64 | 100 |
| Sulfate permease family protein† | *VC_0587* | Q9KUD5 | *OSU_1972* | L1QXE3 | 99 |
| Multidrug resistance protein, putative/ RND multidrug efflux transporter/ Acriflavin resistance protein† | *VC_0164* | Q9KVI2 | *OSU_3387* | L1QSW7 | 99 |
| Succinate dehydrogenase, flavoprotein subunit† | *VC_2089* | Q9KQB1 | *OSU_0312* | L1R1G6 | 100 |
| Peptide ABC transporter, ATP-binding protein/ Peptide transport system ATP-binding protein SapD† | *VC_1683* | Q9KRF9 | *OSU_1953* | L1QXJ4 | 100 |
| Sulfate permease family protein† | *VC_A0103* | Q9KN62 | *OSU_1559* | L1QXS9 | 100 |
| AmpG protein, putative† | *VC_2300* | Q9KPR6 | *OSU_0393* | L1R110 | 99 |
| NupC family protein† | *VC_1953* | Q9KQP5 | *OSU_3100* | L1QUA2 | 99 |
| Putative uncharacterized protein/Putative 3-phenylpropionic acid transporter† | *VC_0302* | Q9KV55 | *OSU_1788* | L1QY91 | 99 |
| Oligopeptide ABC transporter, ATP-binding protein† | *VC_1095* | Q9KT10 | *OSU_2439* | L1QVA7 | 100 |
| SgaT protein/ Ascorbate-specific PTS system, EIIC component† | *VC_A0246* | Q9KMS4 | *OSU_0491* | L1R0Z6 | 100 |
| PTS system, fructose-specific IIABC component† | *VC_1822* | Q9KR25 | *OSU_1264* | L1QYJ7 | 99 |
| PTS system, fructose-specific IIABC component† | *VC_1826* | Q9KR21 | *OSU_1268* | L1QYE9 | 99 |

| | | | | | |
|---|---|---|---|---|---|
| PTS system, sucrose-specific IIBC component† | *VC_A0653* | Q9KLT8 | *OSU_2569* | L1QW91 | 99 |
| PTS system, glucose-specific IIBC component† | *VC_2013* | Q9KQI5 | *OSU_1674* | L1QXA9 | 100 |
| PTS system, trehalose-specific IIBC component† | *VC_0910* | Q9KTJ2 | *OSU_2271* | L1QWI9 | 99 |
| Cytochrome c oxidase, subunit CcoN† | *VC_1442* | Q9KS19 | *OSU_0071* | L1R2B6 | 100 |
| Cytochrome b† | *VC_0574* | Q9KUE7 | *OSU_1984* | L1QXU0 | 100 |
| Ubiquinol-cytochrome c reductase iron-sulfur subunit† | *VC_0573* | Q9KUE8 | *OSU_1985* | L1QWJ5 | 100 |
| Sensor histidine kinase† | *VC_0303* | Q9KV54 | *OSU_1787* | L1QXA3 | 99 |
| TolR membrane protein† | *VC_1838* | Q9KR09 | *OSU_1278* | L1QYF9 | 100 |
| TonB system transport protein ExbB2/ Ferric siderophore transport system, biopolymer transport protein ExbB† | *VC_1546* | Q9KRT9 | *OSU_3209* | L1QT39 | 99 |
| C4-dicarboxylate transport protein† | *VC_1927* | Q9KQS1 | *OSU_3077* | L1QUN1 | 100 |
| Branched chain amino acid transport system II carrier protein† | *VC_0662* | Q9KU61 | *OSU_3014* | L1QUY2 | 99 |
| Spermidine/putrescine ABC transporter, permease protein† | *VC_1427* | Q9KS34 | *OSU_0056* | L1R294 | 100 |
| Spermidine/putrescine ABC transporter, permease protein† | *VC_1426* | Q9KS35 | *OSU_0055* | L1R2W3 | 100 |
| Oligopeptide ABC transporter, permease protein† | *VC_1092* | Q9KT13 | *OSU_2436* | L1QW65 | 100 |
| Peptide ABC transporter, permease protein† | *VC_1681* | Q9KRG1 | *OSU_1955* | L1QXY0 | 99 |
| ABC transporter, permease protein, putative† | *VC_1665* | Q9KRH7 | *OSU_1725* | L1QYK0 | 99 |
| Peptide ABC transporter, permease protein/ (GlcNAc)2 ABC transporter, permease component 2† | *VC_0618* | Q9KUA5 | *OSU_3342* | L1QSN9 | 100 |
| Hemin ABC transporter, permease protein, putative† | *VC_A0914* | Q9KL35 | *OSU_2643* | L1QV06 | 99 |
| Peptide ABC transporter, permease protein/ (GlcNAc)2 ABC transporter, permease component 1† | *VC_0619* | Q9KUA4 | *OSU_3343* | L1QSS9 | 99 |
| Peptide ABC transporter, permease protein† | *VC_0172* | Q9KVH4 | *OSU_3227* | L1QTP2 | 99 |

| | | | | | |
|---|---|---|---|---|---|
| Quaternary ammonium compound-resistance protein SugE† | *VC_1393* | Q9KS66 | *OSU_2314* | L1QVH9 | 85 |
| Glycerol-3-phosphate ABC transporter, permease protein† | *VC_1551* | Q9KRT5 | *OSU_3204* | L1QT34 | 99 |
| Glycerol-3-phosphate ABC transporter, permease protein† | *VC_1550* | Q9KRT6 | *OSU_3205* | L1QT72 | 99 |
| LysE/YggA family protein/ Lysine efflux permease† | *VC_0481* | Q9KUN4 | *OSU_2859* | L1QVI7 | 100 |
| Iron(III) ABC transporter, permease protein/ *Vibrio*bactin and enterobactin ABC transporter, permease protein† | *VC_A0229* | Q9KMU0 | *OSU_0356* | L1R152 | 99 |
| Galactose/methyl galactoside ABC transport system, permease protein MglC† | *VC_1328* | Q9KSD0 | *OSU_0592* | L1R1S3 | 100 |
| Putative threonine efflux protein† | *VC_1939* | Q9KQQ9 | *OSU_3088* | L1QTB2 | 100 |
| Homoserine/homoserine lactone efflux protein† | *VC_0136* | Q9KVK7 | *OSU_3509* | L1QSK1 | 99 |
| Iron(III) ABC transporter, permease protein/ Ferric *Vibrio*bactin, enterobactin transport system, permease protein VctD† | *VC_A0228* | Q9KMU1 | *OSU_0357* | L1R199 | 100 |
| Ribose ABC transporter, permease protein† | *VC_A0129* | Q9KN36 | *OSU_1584* | L1QXU9 | 99 |
| ABC transporter, permease protein, putative† | *VC_1102* | Q9KT03 | *OSU_2446* | L1QW73 | 100 |
| Lysine/cadaverine antiporter membrane protein CadB† | *VC_0280* | Q9KV76 | *OSU_1810* | L1QX58 | 100 |
| Zinc ABC transporter, inner membrane permease protein ZnuB† | *VC_2083* | Q9KQB7 | *OSU_0318* | L1R264 | 100 |
| Transporter, putative† | *VC_0338* | Q9KV20 | *OSU_0802* | L1R029 | 99 |
| Transporter, putative† | *VC_1314* | Q9KSE3 | *OSU_0580* | L1R183 | 99 |
| Sodium/solute symporter, putative/ Acetate permease ActP (Cation/acetate symporter)† | *VC_2705* | Q9KNM7 | *OSU_1256* | L1R002 | 100 |
| Transporter† | *VC_2012* | Q9KQI6 | *OSU_2786* | L1QUC7 | 99 |
| Sodium-dependent transporter† | *VC_1669* | Q9KRH3 | *OSU_1729* | L1QXK2 | 99 |

| | | | | | |
|---|---|---|---|---|---|
| Small-conductance mechanosensitive channel† | *VC_1751* | Q9KR91 | *OSU_3307* | L1QT25 | 99 |
| Tellurite resistance protein† | *VC_A0524* | Q9KM64 | *OSU_3418* | L1QSE4 | 99 |
| Chemotaxis protein PomA/ Flagellar motor rotation protein MotA† | *VC_0892* | Q9KTL0 | *OSU_0288* | L1R237 | 97 |
| Extracellular solute-binding protein, putative† | *VC_0488* | Q9KUM7 | *OSU_2852* | L1QV15 | 99 |
| Multidrug resistance protein D† | *VC_A0083* | Q9KN82 | *OSU_1537* | L1QXY3 | 99 |
| Tellurite resistance protein† | *VC_2323* | Q9KPP3 | *OSU_1024* | L1QZ90 | 100 |
| Extracellular solute-binding protein, family 7† | *VC_1273* | Q9KSI3 | *OSU_0738* | L1R062 | 99 |
| Putative Co/Zn/Cd efflux system membrane fusion protein† | *VC_0913* | Q9KTI9 | *OSU_1334* | L1QYC0 | 99 |
| Oxalate/formate antiporter, putative† | *VC_A0554* | Q9KM35 | *OSU_2882* | L1QU35 | 99 |
| Citrate/sodium symporter/ $Na^+$Citrate $OH^-$ antiporter† | *VC_0795* | Q9KTU3 | *OSU_0157* | L1R1Z8 | 100 |
| Membrane protein† | *VC_1229* | Q9KSM7 | *OSU_0696* | L1R0B8 | 99 |
| Putative uncharacterized protein† | *VC_A0024* | Q9KNE1 | *OSU_1930* | L1QWU8 | 100 |
| Membrane-fusion protein† | *VC_0628* | Q9KU95 | *OSU_3353* | L1QST9 | 99 |
| Putative Co/Zn/Cd efflux system membrane fusion protein† | *VC_1675* | Q9KRG7 | *OSU_1961* | L1QWP1 | 99 |
| Putative Co/Zn/Cd efflux system membrane fusion protein† | *VC_1756* | Q9KR86 | *OSU_3303* | L1QU34 | 95 |
| Type I secretion system, outer membrane component (LapE)† | *VC_1621* | Q9KRL6 | *OSU_2687* | L1QVC0 | 99 |
| Sodium/alanine symporter/ $Na^+$-linked D-alanine glycine permease† | *VC_2356* | Q9KPL1 | *OSU_0093* | L1R2Y0 | 99 |
| Putative uncharacterized protein VCA0047† | *VC_A0047* | Q9KNB8 | *OSU_1504* | L1QXK0 | 99 |
| Putative uncharacterized protein† | *VC_A0181* | Q9KMY5 | *OSU_3476* | L1QTQ5 | 99 |
| Multidrug resistance protein, putative† | *VC_1618* | Q9KRL8 | *OSU_2690* | L1QUF5 | 99 |
| Inner membrane protein YrbG, predicted calcium/sodium:proton antiporter† | *VC_2522* | Q9KP54 | *OSU_1078* | L1QZW4 | 99 |

| | | | | | |
|---|---|---|---|---|---|
| Permease† | *VC_A0904* | Q9KL44 | *OSU_2633* | L1QUZ3 | 99 |
| Permease of the major facilitator superfamily† | *VC_1071* | Q9KT34 | *OSU_2415* | L1QVB3 | 99 |
| Cytochrome b561, putative† | *VC_A0538* | Q9KM51 | *OSU_3432* | L1QTU0 | 99 |
| Small-conductance mechanosensitive channel† | *VC_0265* | Q9KV91 | *OSU_1822* | L1QXD7 | 100 |
| Membrane-fusion protein† | *VC_1563* | Q9KRS3 | *OSU_3189* | L1QT16 | 99 |
| Small-conductance mechanosensitive channel† | *VC_A0817* | Q9KLC9 | *OSU_1330* | L1QYD9 | 100 |
| Putative Co/Zn/Cd efflux system membrane fusion protein† | *VC_1674* | Q9KRG8 | *OSU_1962* | L1QWS8 | 99 |
| Putative Co/Zn/Cd efflux system membrane fusion protein† | *VC_0165* | Q9KVI1 | *OSU_3386* | L1QTF5 | 99 |
| Membrane protein† | *VC_1870* | Q9KQX7 | *OSU_1309* | L1QYN9 | 100 |
| AcrA/AcrE family protein† | *VC_A0639* | Q9KLV2 | *OSU_2555* | L1QUW9 | 99 |
| Sodium/alanine symporter† | *VC_0784* | Q9KTV3 | *OSU_0167* | L1R208 | 100 |
| RND multidrug efflux transporter/ Acriflavin resistance protein† | *VC_0629* | Q9KU94 | *OSU_3354* | L1QTK8 | 99 |
| Permease of the major facilitator superfamily† | *VC_A0753* | Q9KLJ1 | *OSU_2884* | L1QU79 | 99 |
| Sodium/glycine symporter GlyP† | *VC_1422* | Q9KS39 | *OSU_0050* | L1R2U8 | 99 |
| Xanthine/uracil/thiamine/ascorbate permease family protein† | *VC_2278* | Q9KPT4 | *OSU_0414* | L1R174 | 99 |
| Phosphate ABC transporter, permease protein† | *VC_A0071* | Q9KN94 | *OSU_1525* | L1QXR6 | 99 |
| L-lysine permease† | *VC_0191* | Q9KVF5 | *OSU_3234* | L1QU68 | 97 |
| Ferric *Vibrio*bactin ABC transporter, permease protein† | *VC_0777* | H9L4Q8 | *OSU_0173* | L1R362 | 99 |
| L-lysine permease† | *VC_A1000* | Q9KKV0 | *OSU_1735* | L1QX50 | 99 |
| Putative threonine efflux protein† | *VC_1421* | Q9KS40 | *OSU_0049* | L1R236 | 99 |
| Ferric *Vibrio*bactin ABC transporter, permease protein† | *VC_0778* | H9L4Q0 | *OSU_0172* | L1R213 | 97 |
| Putative uncharacterized protein VCA0846† | *VC_A0846* | Q9KLA0 | *OSU_2139* | L1QWE3 | 100 |
| Proton/glutamate symporter† | *VC_1168* | Q9KST8 | *OSU_2748* | L1QUA3 | 100 |
| Na+/H+ antiporter† | *VC_A1015* | H9L4S4 | *OSU_1365* | L1QY89 | 99 |

| | | | | | |
|---|---|---|---|---|---|
| ABC transporter, ATP-binding protein† | *VC_1499* | Q9KRY4 | *OSU_0909* | L1R0X0 | 99 |
| NAD(P) transhydrogenase subunit alpha† | *VC_A0563* | Q9KM26 | *OSU_3533* | L1QS96 | 99 |
| ABC-type antimicrobial peptide transport system, permease component† | *VC_1567* | Q9KRR9 | *OSU_3186* | L1QTY3 | 99 |
| ABC-type antimicrobial peptide transport system, permease component† | *VC_1566* | Q9KRS0 | *OSU_3187* | L1QTD1 | 100 |
| ABC-type antimicrobial peptide transport system, permease component† | *VC_2554* | Q9KP25 | *OSU_1107* | L1QYY8 | 99 |
| Putative TEGT family carrier/transport protein† | *VC_1358* | Q9KSA1 | *OSU_2347* | L1QVX1 | 100 |
| Permease of the major facilitator superfamily† | *VC_A0085* | Q9KN80 | *OSU_1539* | L1QXN5 | 99 |
| Cytochrome c-type biogenesis protein CcmD, interacts with CcmCE / Heme exporter protein D† | *VC_2054* | Q9KQE6 | *OSU_0998* | L1R0P6 | 99 |
| Putative low-affinity inorganic phosphate transporter† | *VC_2442* | Q9KPD0 | *OSU_2029* | L1QWJ3 | 100 |
| L-lactate permease, putative† | *VC_A0983* | Q9KKW7 | *OSU_1764* | L1QYI3 | 99 |
| Putative lipoprotein YajG† | *VC_2298* | Q9KPR8 | *OSU_0395* | L1R1W4 | 100 |
| Crossover junction endodeoxyribonuclease RuvC§ | *ruvC* | Q9KR00 | *ruvC* | L1QYR7 | 99 |
| Holliday junction ATP-dependent DNA helicase RuvA§ | *ruvA* | Q9KR01 | *ruvA* | L1QZA4 | 99 |
| Holliday junction ATP-dependent DNA helicase RuvB§ | *ruvB* | Q9KR02 | *ruvB* | L1QYL6 | 99 |
| Phosphoglycolate phosphatase§ | *gph* | Q9KNV6 | *OSU_1177* | L1QZ52 | 99 |
| DNA ligase§ | *ligA* | Q9KTD1 | *ligA* | L1QX70 | 99 |
| Formamidopyrimidine-DNA glycosylase§ | *mutM* | Q9KVC5 | *mutM* | L1QU94 | 100 |
| Uracil-DNA glycosylase§ | *ung* | Q9KPK8 | *ung* | L1R2E3 | 99 |
| UvrABC system protein A§ | *uvrA* | Q9KUW5 | *uvrA* | L1QY95 | 99 |
| UvrABC system protein C§ | *uvrC* | Q9KSP2 | *uvrC* | L1R101 | 99 |
| DNA mismatch repair protein MutH§ | *mutH* | Q9KU56 | *mutH* | L1QTQ3 | 99 |
| DNA recombination protein RmuC homolog§ | *rmuC* | Q9KVQ7 | *OSU_2198* | L1QW24 | 99 |
| DNA repair protein RecN§ | *recN* | P0C6Q4 | *OSU_0794* | L1R0S5 | 99 |
| DNA replication and repair protein RecF§ | *recF* | Q9KVX4 | *recF* | L1R1N1 | 99 |

| | | | | | |
|---|---|---|---|---|---|
| Probable endonuclease 4§ | *nfo* | Q9KPK7 | *nfo* | L1R1T9 | 99 |
| Exodeoxyribonuclease 7 large subunit§ | *xseA* | Q9KTW4 | *xseA* | L1R2P1 | 99 |
| Exodeoxyribonuclease 7 small subunit§ | *xseB* | Q9KTL1 | *xseB* | L1R1E6 | 100 |
| DNA adenine methylasev | *dam* | P0C6Q8 | *OSU_1179* | L1QYV7 | 100 |
| Protein RecA§ | *recA* | P45383 | *recA* | L1QUC9 | 99 |
| LexA repressor§ | *lexA* | Q9KVP9 | *lexA* | L1QWS7 | 100 |
| Single-stranded DNA-binding protein§ | *ssb* | Q9KUW2 | *OSU_1451* | L1QY38 | 99 |
| DNA mismatch repair protein MutL§ | *mutL* | Q9KV13 | *mutL* | L1R0Q1 | 99 |
| DNA mismatch repair protein MutS§ | *mutS* | Q9KUI6 | *mutS* | L1QU15 | 99 |
| DNA repair protein RecO§ | *recO* | Q9KPB4 | *recO* | L1QWT2 | 99 |
| Regulatory protein RecX§ | *recX* | Q56647 | *recX* | L1QUW6 | 97 |
| Recombination protein RecR§ | *recR* | Q9KT49 | *recR* | L1QV98 | 100 |
| ATP-dependent RNA helicase RhlB§ | *rhlB* | Q9KV52 | *rhlB* | L1QX34 | 100 |
| RNA polymerase-associated protein RapA§ | *rapA* | Q9KP70 | *rapA* | L1QX27 | 99 |
| tRNA(Met) cytidine acetyltransferase TmcA§ | *tmcA* | Q9KKJ5 | *tmcA* | L1QV40 | 99 |
| Transcription termination factor Rho§ | *rho* | Q9KV50 | *rho* | L1QY86 | 100 |
| Cryptochrome DASH§ | *cry1* | Q9KR33 | *OSU_1627* | L1QXT9 | 97 |
| Chromosomal replication initiator protein DnaA§ | *dnaA* | Q9KVX6 | *dnaA* | L1R154 | 99 |
| DNA polymerase III subunit alpha§ | *dnaE* | P52022 | *OSU_3150* | L1QTG4 | 99 |
| DNA polymerase III subunit beta§ | *dnaN* | Q9KVX5 | *OSU_0678* | L1R0J4 | 99 |
| Primosomal replication protein n§ | *priB* | Q9KUZ1 | *OSU_0448* | L1R2A8 | 100 |
| DNA topoisomerase 1§ | *topA* | Q9KRB2 | *OSU_3325* | L1QSW2 | 99 |
| DNA topoisomerase 3§ | *topB* | Q9KQF5 | *OSU_1010* | L1QZJ7 | 99 |
| DNA gyrase subunit A§ | *gyrA* | Q9KSJ8 | *gyrA* | L1R046 | 99 |
| DNA gyrase subunit B§ | *gyrB* | Q9KVX3 | *gyrB* | L1R0B1 | 99 |
| Extracellular deoxyribonuclease Dns/Endonuclease I§ | *dns* | P08038 | *OSU_1893* | L1QY21 | 99 |
| Chromosome partition protein MukB§ | *mukB* | Q9KRC8 | *mukB* | L1QZJ8 | 99 |
| Chromosome partition protein MukE§ | *mukE* | Q9KRC7 | *mukE* | L1QY96 | 99 |

| | | | | | |
|---|---|---|---|---|---|
| Chromosome partition protein MukF§ | *mukF* | Q9KRC6 | *mukF* | L1QYF1 | 99 |
| DNA-binding protein Fis§ | *fis* | P64127 | *fis* | L1QXB2 | 100 |
| Regulatory protein LuxO§ | *luxO* | Q9KT84 | *OSU_0967* | L1QZS0 | 100 |
| DNA-binding protein HU-alpha§ | *hupA* | Q9KV83 | *OSU_1817* | L1QXD1 | 100 |
| DNA-binding protein HU-beta§ | *hupB* | Q9KQS9 | *OSU_3069* | L1QTC5 | 100 |
| Integration host factor alpha subunit§ | *ihfA* | Q9KSN4 | *ihfA* | L1R0B4 | 100 |
| Integration host factor beta subunit§ | *ihfB* | Q9KQT4 | *ihfB* | L1QSP7 | 100 |
| Tryptophan--tRNA ligase§ | *trpS* | Q9KNV7 | *trpS* | L1QZR4 | 99 |
| Phosphoribosylformylglycinamidine cyclo-ligase§ | *purM* | Q9KPY6 | *purM* | L1QV35 | 99 |
| Methionine--tRNA ligase§ | *metG* | Q9KT69 | *metG* | L1QV76 | 99 |
| Glutamate-ammonia-ligase adenylyltransferase§ | *glnE* | Q9KPD4 | *glnE* | L1QWI7 | 99 |
| Lipoate-protein ligase A§ | *lplA* | Q9KS71 | *OSU_2320* | L1QVM5 | 99 |
| Cysteine--tRNA ligase§ | *cysS* | Q9KQZ9 | *cysS* | L1QZS1 | 99 |
| Glutamate--tRNA ligase§ | *gltX* | P0C6Q1 | *gltX* | L1R0R5 | 100 |
| Leucine--tRNA ligase§ | *leuS* | Q9KTE6 | *leuS* | L1R1N8 | 99 |
| Glutamine--tRNA ligase§ | *glnS* | Q9KTA6 | *glnS* | L1QUH7 | 100 |
| Aspartate--tRNA ligase§ | *aspS* | Q9KSU0 | *aspS* | L1QV64 | 99 |
| Lysine--tRNA ligase§ | *lysS* | Q9KU60 | *lysS* | L1QTP7 | 100 |
| Asparagine--tRNA ligase§ | *asnS* | Q9KSF9 | *asnS* | L1QVF2 | 99 |
| Arginine--tRNA ligase§ | *argS* | Q9KQC6 | *argS* | L1QZC0 | 99 |
| Threonine--tRNA ligase§ | *thrS* | Q9KMN7 | *thrS* | L1R261 | 99 |
| Glutamine synthetase§ | *glnA* | Q9KNJ2 | *OSU_0009* | L1R2Z1 | 100 |
| Argininosuccinate synthase§ | *argG* | Q9KNT8 | *argG* | L1QYX3 | 99 |
| CTP synthase§ | *pyrG* | Q9KPC4 | *pyrG* | L1QXC4 | 99 |
| UDP-N-acetylmuramate-L-alanine ligase§ | *murC* | Q9KPG8 | *murC* | L1R320 | 99 |
| Tyrosine--tRNA ligase 2§ | *tyrS2* | Q9KU92 | *tyrS* | L1R1Y4 | 99 |
| Glutamate--cysteine ligase§ | *gshA* | Q9KUG5 | *gshA* | L1QWP0 | 99 |
| Phenylalanine--tRNA ligase alpha subunit§ | *pheS* | Q9KSN7 | *pheS* | L1R009 | 99 |

| | | | | | |
|---|---|---|---|---|---|
| Glycine--tRNA ligase alpha subunit§ | *glyQ* | Q9KVW7 | *glyQ* | L1R1I0 | 100 |
| Glycine--tRNA ligase beta subunit§ | *glyS* | Q9KVW8 | *glyS* | L1R1E2 | 99 |
| Histidine--tRNA ligase§ | *hisS* | Q9KTX0 | *hisS* | L1R233 | 100 |
| Pantothenate synthetase§ | *panC* | Q9KUD1 | *panC* | L1R0P9 | 99 |
| D-alanine--D-alanine ligase§ | *ddl* | Q9KM17 | *ddl* | L1R0E4 | 99 |
| UDP-N-acetylmuramoylalanine--D-glutamate ligase§ | *murD* | Q9KPG5 | *murD* | L1R2I6 | 99 |
| *Vibrio*bactin-specific 2,3-dihydroxybenzoate-AMP ligase§ | *vibE* | O07899 | *OSU_0178* | L1R369 | 99 |
| Alanine--tRNA ligase§ | *alaS* | Q56648 | *alaS* | L1QU49 | 99 |
| Isoleucine--tRNA ligase§ | *ileS* | Q9KU47 | *ileS* | L1QTM1 | 99 |
| Proline--tRNA ligase§ | *proS* | Q9KTM7 | *proS* | L1R223 | 99 |
| Elongation factor P--(R)-beta-lysine ligase§ | *epmA* | Q9KNS6 | *epmA* | L1QZ34 | 99 |
| Succinyl-CoA ligase (ADP-forming) subunit beta§ | *sucC* | Q9KQB5 | *sucC* | L1R1D2 | 100 |
| Serine-tRNA ligase§ | *serS* | Q9KSZ6 | *serS* | L1QWL9 | 99 |
| Phenylalanine--tRNA ligase beta subunit§ | *pheT* | Q9KSN6 | *pheT* | L1R070 | 99 |
| Phosphoribosylamine--glycine ligase§ | *purD* | Q9KV81 | *purD* | L1QX62 | 99 |
| Acetyl-coenzyme A synthetase§ | *acsA* | Q9KV59 | *acsA* | L1QXU2 | 99 |
| UDP-N-acetylmuramoyl-L-alanyl-D-glutamate--2,6-diaminopimelate ligase§ | *murE* | Q9X6N4 | *murE* | L1R1Q3 | 99 |
| Fatty acid metabolism regulator protein§ | *fadR* | Q9KQU8 | *fadR* | L1QTF9 | 100 |
| Guanosine-3',5'-bis(diphosphate) 3'-pyrophosphohydrolase§ | *spoT* | Q9KNM2 | *OSU_1260* | L1QZB2 | 99 |
| Succinylglutamate desuccinylase§ | *astE* | Q9KSL4 | *astE* | L1R027 | 99 |
| Formimidoylglutamase§ | *hutG* | Q9KSQ2 | *hutG* | L1R112 | 94 |
| Orotidine 5'-phosphate decarboxylase§ | *pyrF* | Q9KQT7 | *pyrF* | L1QSY0 | 99 |
| 3-oxoacyl-(acyl-carrier-protein) synthase 2§ | *fabF* | Q9KQH9 | *OSU_1680* | L1QXG5 | 99 |
| 3-oxoacyl-(acyl-carrier-protein) reductase FabG§ | *fabG* | Q9KQH7 | *OSU_1682* | L1QXM3 | 100 |
| Holo-(acyl-carrier-protein) synthase§ | *acpS* | Q9KPB6 | *acpS* | L1QWK4 | 100 |

| | | | | | |
|---|---|---|---|---|---|
| Phosphoheptose isomerase§ | *gmhA* | Q9KPY2 | *gmhA* | L1R028 | 100 |
| Orotate phosphoribosyltransferase§ | *pyrE* | Q9KVD5 | *pyrE* | L1QT70 | 99 |
| D-glycero-beta-D-manno-heptose-1,7-bisphosphate 7-phosphatase§ | *gmhB* | Q9KTJ4 | *OSU_2273* | L1QWZ7 | 100 |
| Carbamoyl-phosphate synthase large chain§ | *carB* | Q9KPH9 | *carB* | L1R2H0 | 100 |
| N-acetylglucosamine-6-phosphate deacetylase§ | *nagA* | O32445 | *OSU_2708* | L1QUT8 | 99 |
| Coenzyme A biosynthesis bifunctional protein CoaBC§ | *coaBC* | Q9KVD1 | *OSU_3252* | L1QTR6 | 99 |
| Uridine kinase§ | *udk* | Q9KT67 | *udk* | L1QVG9 | 100 |
| Adenine phosphoribosyltransferase§ | *apt* | Q9KT52 | *apt* | L1QVI5 | 100 |
| Arginine deiminase§ | *arcA* | Q9KUU2 | *arcA* | L1QYX2 | 99 |
| Adenylyl-sulfate kinase§ | *cysC* | Q9KP21 | *cysC* | L1QYU0 | 99 |
| Glucans biosynthesis protein G§ | *opgG* | Q9KSG8 | *opgG* | L1R0G3 | 99 |
| Methylisocitrate lyase§ | *prpB* | Q9KSC2 | *OSU_2367* | L1QVZ2 | 99 |
| Galactokinase§ | *galK* | Q9KRP1 | *galK* | L1QZF7 | 99 |
| Phosphate acyltransferase§ | *plsX* | Q9KQH4 | *plsX* | L1QXH1 | 100 |
| Glycerol-3-phosphate acyltransferase§ | *plsY* | Q9KUJ7 | *plsY* | L1QUY3 | 99 |
| Acetyl-coenzyme A carboxylase carboxyl transferase subunit alpha§ | *accA* | Q9KPW8 | *accA* | L1QU31 | 99 |
| Acyl carrier protein (ACP)§ | *acpP* | Q9KQH8 | *acpP* | L1QY72 | 100 |
| 3-ketoacyl-CoA thiolase§ | *fadA* | Q9KNI0 | *fadA* | L1R0D3 | 99 |
| 3-ketoacyl-CoA thiolase§ | *fadI* | Q9KT59 | *fadI* | L1QV87 | 99 |
| Glycerol-3-phosphate acyltransferase (GPAT)§ | *plsB* | Q9KVP8 | *plsB* | L1QW12 | 99 |
| Phosphatidylserine decarboxylase proenzyme§ | *psd* | Q9KV19 | *psd* | L1R0M2 | 99 |
| Anhydro-N-acetylmuramic acid kinase§ | *anmK* | Q9KU38 | *anmK* | L1QU07 | 99 |
| Glucose-1-phosphate adenylyltransferase 1§ | *glgC1* | Q9KRB5 | *glgC* | L1QZ56 | 100 |
| Glucose-1-phosphate adenylyltransferase 2§ | *glgC2* | Q9KLP4 | *glgC* | L1QTC2 | 99 |
| Glycerol kinase§ | *glpK* | Q9KLJ9 | *glpK* | L1QTZ9 | 99 |
| D-ribose pyranase§ | *rbsD* | Q9KN38 | *rbsD* | L1QY55 | 99 |

| | | | | | |
|---|---|---|---|---|---|
| HTH-type transcriptional regulator MalT§ | *malT* | Q9KNF3 | *malT* | L1QUN0 | 99 |
| Fructose-1,6-bisphosphatase class 1§ | *fbp* | Q9KP35 | *fbp* | L1QYX9 | 100 |
| Bifunctional protein FolD§ | *folD* | Q9KQQ6 | *folD* | L1QUP8 | 100 |
| Adenylate kinase§ | *adk* | Q9KTB7 | *adk* | L1QUG6 | 100 |
| N-acetyl-D-glucosamine kinase§ | *nagK* | Q9KRV2 | *nagK* | L1R209 | 99 |
| Bifunctional purine biosynthesis protein PurH§ | *purH* | Q9KV80 | *purH* | L1QX20 | 99 |
| HTH-type transcriptional repressor PurR§ | *purR* | Q9KRC1 | *purR* | L1QYF5 | 100 |
| Thymidylate synthase§ | *thyA* | O66108 | *thyA* | L1QTZ3 | 99 |
| Lipase§ | *lipA* | P15493 | *OSU_0768* | L1R0U3 | 99 |
| Adenosine deaminase§ | *add* | Q9KNI7 | *add* | L1R2Y7 | 99 |
| N-succinylglutamate 5-semialdehyde dehydrogenase§ | *astD* | Q9KNW4 | *astD* | L1QZ43 | 99 |
| Carbamoyl-phosphate synthase small chain§ | *carA* | Q9KPH8 | *carA* | L1R1W5 | 99 |
| Sulfate adenylyltransferase subunit 2§ | *cysD* | Q9KP19 | *cysD* | L1QYZ3 | 99 |
| Phosphoadenosine phosphosulfate reductase§ | *cysH* | Q9KUX2 | *cysH* | L1R0W9 | 97 |
| Sulfate adenylyltransferase subunit 1§ | *cysN* | Q9KP20 | *cysN* | L1QZI3 | 99 |
| Uroporphyrinogen decarboxylase§ | *hemE* | Q9KV26 | *hemE* | L1R153 | 100 |
| GTPase Era§ | *era* | Q9KPB3 | *era* | L1QXS1 | 96 |
| 3-hydroxyacyl-(acyl-carrier-protein) dehydratase FabZ§ | *fabZ* | Q9KPW3 | *fabZ* | L1QU36 | 99 |
| GTP cyclohydrolase 1§ | *folE* | Q9KLX5 | *folE* | L1R156 | 100 |
| Glutamine--fructose-6-phosphate aminotransferase (isomerizing)§ | *glmS* | Q9KUM8 | *glmS* | L1QUH4 | 99 |
| Hydroxyacylglutathione hydrolase§ | *gloB* | Q9KPX6 | *gloB* | L1QZH9 | 100 |
| Anaerobic glycerol-3-phosphate dehydrogenase subunit B§ | *glpB* | Q9KLJ6 | *glpB* | L1QUS7 | 99 |
| Guanosine-5'-triphosphate,3'-diphosphate pyrophosphatase§ | *gppA* | Q9KV53 | *gppA* | L1QXT7 | 99 |
| Glutamate-1-semialdehyde 2,1-aminomutase§ | *hemL* | Q9KU97 | *hemL* | L1QT15 | 100 |
| Glutathione synthetase§ | *gshB* | Q9KUP7 | *gshB* | L1QWW9 | 99 |

| | | | | | |
|---|---|---|---|---|---|
| GMP synthase (glutamine-hydrolyzing)§ | *guaA* | Q9KTW2 | *guaA* | L1R1V1 | 99 |
| Porphobilinogen deaminase§ | *hemC* | Q9KVM1 | *hemC* | L1QT69 | 99 |
| Histidine ammonia-lyase§ | *hutH* | Q9KSQ4 | *hutH* | L1QZV1 | 100 |
| Imidazolonepropionase§ | *hutI* | Q9KSQ1 | *hutI* | L1QZZ9 | 99 |
| Urocanate hydratase§ | *hutU* | Q9KSQ3 | *hutU* | L1QZP5 | 99 |
| Probable lactoylglutathione lyase§ | *gloA* | Q9KT93 | *OSU_2725* | L1QUD9 | 100 |
| Acyl-(acyl-carrier-protein)--UDP-N-acetylglucosamine O-acyltransferase§ | *lpxA* | Q9KPW4 | *lpxA* | L1QTA0 | 100 |
| Lipid-A-disaccharide synthase§ | *lpxB* | Q9KPW5 | *lpxB* | L1QT61 | 99 |
| UDP-3-O-(3-hydroxymyristoyl) N-acetylglucosamine deacetylase§ | *lpxC* | Q9KPH2 | *lpxC* | L1R1P4 | 100 |
| UDP-3-O-acylglucosamine N-acyltransferase§ | *lpxD* | Q9KPW2 | *lpxD* | L1QTG7 | 99 |
| UDP-2,3-diacylglucosamine hydrolase§ | *lpxH* | Q9KQZ7 | *lpxH* | L1QYM1 | 99 |
| Nucleoside diphosphate kinase§ | *ndk* | Q9KTX4 | *ndk* | L1R2Q4 | 100 |
| Glucans biosynthesis glucosyltransferase H§ | *opgH* | Q9KSG9 | *OSU_0745* | L1R113 | 99 |
| Phosphate acetyltransferase§ | *pta* | Q9KT08 | *OSU_2441* | L1QW69 | 100 |
| Phosphocarrier protein NPr§ | *npr* | Q9KP46 | *OSU_1088* | L1QZX4 | 100 |
| Phosphoribosylformylglycinamidine synthase§ | *purL* | Q9KTN2 | *purL* | L1QZX1 | 99 |
| Phosphoribosylaminoimidazole-succinocarboxamide synthase§ | *purC* | Q9KSR6 | *OSU_0865* | L1R0K4 | 99 |
| Aspartate carbamoyltransferase§ | *pyrB* | Q9KP66 | *pyrB* | L1QW68 | 100 |
| Dihydroorotase§ | *pyrC* | Q9KL24 | *pyrC* | L1QVM9 | 97 |
| Dihydroorotate dehydrogenase (quinone)§ | *pyrD* | Q9KRZ2 | *pyrD* | L1QZQ2 | 99 |
| Uridylate kinase§ | *pyrH* | Q9KPV4 | *pyrH* | L1QTB0 | 100 |
| 7-cyano-7-deazaguanine synthase§ | *queC* | Q9KS93 | *queC* | L1QVK4 | 99 |
| Thymidine phosphorylase§ | *deoA* | Q9KPL8 | *deoA* | L1R2D3 | 99 |
| Uracil phosphoribosyltransferase§ | *upp* | Q9KPY7 | *upp* | L1QUB2 | 99 |
| Xanthine phosphoribosyltransferase§ | *gpt* | Q9KPT5 | *gpt* | L1R1X8 | 100 |

| | | | | | |
|---|---|---|---|---|---|
| Glycerol-3-phosphate dehydrogenase (NAD(P)+)§ | *gpsA* | Q9KNT0 | *gpsA* | L1QZ74 | 99 |
| Acetyl-coenzyme A carboxylase carboxyl transferase subunit beta§ | *accD* | Q9KTA3 | *accD* | L1QUD0 | 99 |
| 3-hydroxydecanoyl-(acyl-carrier-protein) dehydratase§ | *fabA* | Q9KS00 | *fabA* | L1QZJ1 | 100 |
| 3-oxoacyl-(acyl-carrier-protein) synthase 3 protein 1§ | *fabH1* | Q9KQH5 | *fabH* | L1QXB9 | 99 |
| 3-oxoacyl-(acyl-carrier-protein) synthase 3 protein 2§ | *fabH2* | Q9KLJ3 | *fabH* | L1QTV0 | 99 |
| Fatty acid oxidation complex subunit alpha§ | *fadB* | Q9KNI1 | *fadB* | L1R078 | 99 |
| Fatty acid oxidation complex subunit alpha§ | *fadJ* | Q9KT58 | *fadJ* | L1QW30 | 99 |
| N-acetylmuramic acid 6-phosphate etherase 1§ | *murQ1* | Q9KVE0 | *murQ* | L1QTQ7 | 97 |
| N-acetylmuramic acid 6-phosphate etherase 2§ | *murQ2* | Q9KU39 | *murQ* | L1QUL0 | 100 |
| 1,4-alpha-glucan branching enzyme GlgB§ | *glgB* | Q9KNE8 | *glgB* | L1QV28 | 99 |
| Glucosamine-6-phosphate deaminase§ | *nagB* | Q9KKS5 | *nagB* | L1QY42 | 100 |
| Serine hydroxymethyltransferase 1§ | *glyA1* | Q9KTG1 | *glyA* | L1QVA9 | 100 |
| Serine hydroxymethyltransferase 2§ | *glyA2* | Q9KMP4 | *glyA* | L1R0X8 | 99 |
| Fumarate and nitrate reduction regulatory protein§ | *fnr* | P0C6D0 | *OSU_0063* | L1R222 | 100 |
| Glutamyl-tRNA reductase§ | *hemA* | Q9KQ24 | *hemA* | L1QUI1 | 100 |
| Sulfite reductase (NADPH) hemoprotein beta-component§ | *cysI* | Q9KUX3 | *cysI* | L1R116 | 99 |
| Sulfite reductase (NADPH) flavoprotein alpha-component§ | *cysJ* | Q9KUX4 | *cysJ* | L1R1S8 | 99 |
| S-adenosylmethionine synthase§ | *metK* | Q9KUP3 | *metK* | L1QXM8 | 99 |
| Membrane-bound lytic murein transglycosylase F§ | *mltF* | Q9KTN5 | *mltF* | L1R072 | 100 |
| Periplasmic nitrate reductase§ | *napA* | Q9KLR4 | *napA* | L1R1H7 | 99 |
| Adenylosuccinate synthetase§ | *purA* | Q9KNX8 | *purA* | L1QYT2 | 99 |
| Phosphoribosylglycinamide formyltransferase 2§ | *purT* | Q9KSM8 | *purT* | L1R0W0 | 99 |
| Lipase chaperone§ | *lifO* | O07350 | *lifO* | L1R0A3 | 99 |
| Fumarate hydratase class II§ | *fumC* | Q9KRR3 | *fumC* | L1R0K5 | 99 |
| Inosine-5'-monophosphate dehydrogenase§ | *guaB* | Q9KTW3 | *guaB* | L1R1Y8 | 99 |

| | | | | | |
|---|---|---|---|---|---|
| Phosphoheptose isomerase§ | *gmhA* | Q9KUE2 | *gmhA* | L1QXT6 | 100 |
| Glucosamine-6-phosphate deaminase subunit§ | *nagB* | E5EUX8 | *nagB* | L1QY42 | 100 |
| Putative phosphatase YieH§ | *VC_A0798* | Q9KLE8 | *OSU_0613* | L1R0H0 | 99 |
| DNA polymerase I/DNA polymerase II§ | *VC_1212* | Q9KSP4 | *OSU_0844* | L1QZU3 | 99 |
| DNA-3-methyladenine glycosylase§ | *VC_1672* | Q9KRH0 | *OSU_1964* | L1QX08 | 99 |
| Endonuclease III§ | *VC_1011* | Q9KT92 | *OSU_2726* | L1QUI0 | 99 |
| Exodeoxyribonuclease I/Exodeoxyribonuclease III§ | *VC_1234* | Q9KSM2 | *OSU_0700* | L1R0W4 | 99 |
| Single-stranded-DNA-specific exonuclease RecJ§ | *VC_2417* | Q9KPF1 | *OSU_0146* | L1R2J9 | 99 |
| DNA repair protein RadA/RecA protein§ | *VC_2343* | Q9KPM4 | *OSU_0373* | L1R207 | 99 |
| DNA repair protein RadC§ | *VC_0217* | Q9KVC9 | *OSU_3254* | L1QU89 | 99 |
| DNA-damage-inducible protein J§ | *VC_0090* | Q9KVQ1 | *OSU_2191* | L1QX80 | 96 |
| Exodeoxyribonuclease III§ | *VC_1860* | Q9KQY7 | *OSU_1298* | L1QYH9 | 99 |
| Exonuclease SbcD§ | *VC_A0520* | Q9KM68 | *OSU_3414* | L1QSI3 | 99 |
| Methylated-DNA--protein-cysteine methyltransferase§ | *VC_A1017* | Q9KKT3 | *OSU_1367* | L1QYE7 | 99 |
| Single-stranded DNA-binding protein§ | *VC_2735* | Q9KNK3 | *OSU_0019* | L1R2Z9 | 99 |
| ATP-dependent helicase DinG/Rad3§ | *VC_1855* | Q9KQZ2 | *OSU_1294* | L1QYM4 | 99 |
| ATP-dependent helicase DinG/Rad3§ | *VC_1990* | Q9KQK8 | *OSU_3130* | L1QUF2 | 99 |
| DinG family ATP-dependent helicase YoaA§ | *VC_1598* | Q9KRN8 | *OSU_1053* | L1R0M3 | 99 |
| ATP-dependent DNA helicase Rep§ | *VC_0167* | Q9KVH9 | *OSU_3384* | L1QSI7 | 99 |
| DNA helicase IV§ | *VC_A0717* | Q9KLM6 | *OSU_1643* | L1QYC2 | 100 |
| DNA helicase IV§ | *VC_1636* | Q9KRK4 | *OSU_0755* | L1R1A9 | 99 |
| DNA helicase IV§ | *VC_A0061* | Q9KNA4 | *OSU_1517* | L1QXW0 | 99 |
| ATP-dependent DNA helicase UvrD/PcrA§ | *VC_0190* | Q9KVF6 | *OSU_3233* | L1QT55 | 99 |
| ADA regulatory protein§ | *VC_A1018* | Q9KKT2 | *OSU_1368* | L1QZE1 | 99 |
| ATP-dependent DNA helicase RecG§ | *VC_2711* | Q9KNM1 | *OSU_1261* | L1R015 | 99 |
| ATP-dependent DNA helicase RecQ§ | *VC_0196* | Q9KVF0 | *OSU_3239* | L1QU73 | 99 |
| DNA polymerase III chi subunit§ | *VC_2502* | Q9KP74 | *OSU_2103* | L1QWF9 | 99 |
| DNA polymerase III delta prime subunit§ | *VC_2015* | Q9KQI3 | *OSU_1676* | L1QY65 | 99 |

| | | | | | |
|---|---|---|---|---|---|
| DNA polymerase III delta subunit§ | *VC_0953* | Q9KTE9 | *OSU_0238* | L1R1G0 | 99 |
| DNA polymerase III epsilon subunit§ | *VC_2233* | Q9KPX9 | *OSU_1058* | L1QZ82 | 99 |
| DNA polymerase III epsilon subunit§ | *VC_1290* | Q9KSG6 | *OSU_0748* | L1R073 | 97 |
| DNA polymerase III epsilon subunit§ | *VC_0299* | Q9KV58 | *OSU_1790* | L1QX40 | 99 |
| DNA polymerase III psi subunit§ | *VC_0656* | Q9KU67 | *OSU_3008* | L1QTX7 | 97 |
| DNA polymerase III subunits gamma and tau§ | *VC_1054* | Q9KT51 | *OSU_2398* | L1QWG9 | 99 |
| Helicase PriA essential for oriC/DnaA-independent DNA replication/Primosomal replication protein N§ | *VC_2678* | Q9KNQ3 | *OSU_1229* | L1QZ08 | 99 |
| Primosomal replication protein N prime prime/Primosomal replication protein N§ | *VC_1857* | Q9KQZ0 | *OSU_1296* | L1QYS9 | 99 |
| Replicative DNA helicase§ | *VC_0371* | Q9KUY7 | *OSU_0444* | L1R0Y4 | 100 |
| Transcription-repair coupling factor§ | *VC_1886* | Q9KQW2 | *OSU_1324* | L1QYQ4 | 99 |
| Topoisomerase IV subunit A§ | *VC_2430* | Q9KPE0 | *OSU_2016* | L1QWQ7 | 99 |
| Topoisomerase IV subunit B§ | *VC_2431* | Q9KPD9 | *OSU_2017* | L1QXP9 | 99 |
| Protein involved in catabolism of external DNA§ | *VC_0187* | Q9KVF9 | *OSU_3231* | L1QSX0 | 99 |
| Extracellular nuclease-related protein§ | *VC_2621* | Q9KNV9 | *OSU_1174* | L1QYV3 | 99 |
| Putative uncharacterized protein§ | *VC_1722* | Q9KRC0 | *OSU_1436* | L1QZ50 | 99 |
| Phosphoglycerate transport system transcriptional regulatory protein PgtA§ | *VC_A0704* | Q9KLN9 | *OSU_1654* | L1QXS4 | 100 |
| Nitrogen regulation protein NR(I)§ | *VC_2749* | Q9KNI9 | *OSU_0006* | L1R3G3 | 100 |
| C4-dicarboxylate transport transcriptional regulatory protein§ | *VC_1926* | Q9KQS2 | *OSU_3076* | L1QTK0 | 99 |
| C4-dicarboxylate transport transcriptional regulatory protein§ | *VC_A0142* | Q9KN24 | *OSU_1596* | L1QYS2 | 99 |
| Putative uncharacterized protein§ | *VC_2014* | Q9KQI4 | *OSU_1675* | L1QXF9 | 99 |
| Putative uncharacterized protein§ | *VC_2353* | Q9KPL4 | *OSU_0090* | L1R1P5 | 99 |
| UDP-N-acetylmuramoyl-tripeptide--D-alanyl-D-alanine ligase§ | *VC_2405* | Q9KPG3 | *OSU_0138* | L1R328 | 99 |

| | | | | | |
|---|---|---|---|---|---|
| DNA ligase§ | *VC_1542* | Q9KRU2 | *OSU_0956* | L1R0B2 | 99 |
| UDP-N-acetylmuramate:L-alanyl-gamma-D-glutamyl-meso-diaminopimelate ligase§ | *VC_2542* | Q9KP37 | *OSU_1096* | L1QZG6 | 99 |
| Succinyl-CoA ligase (ADP-forming) subunit alpha§ | *VC_2084* | Q9KQB6 | *OSU_0317* | L1R1H1 | 99 |
| Alpha-acetolactate decarboxylase§ | *VC_1589* | Q9KRP7 | *OSU_1044* | L1QZA5 | 100 |
| Probable sucrose-6-phosphate hydrolase§ | *VC_A0655* | Q9KLT6 | *OSU_2571* | L1QV09 | 97 |
| Citrate synthase§ | *VC_2092* | Q9KQA8 | *OSU_0309* | L1R1K4 | 100 |
| Aconitate hydratase 2§ | *VC_0604* | Q9KUB8 | *OSU_1066* | L1QZI5 | 100 |
| Galactose-1-phosphate uridylyltransferase§ | *VC_1596* | Q9KRP0 | *OSU_1051* | L1R064 | 99 |
| Phosphorylase§ | *VC_A0013* | Q9KNF1 | *OSU_2928* | L1QTT9 | 99 |
| Cardiolipin synthase§ | *VC_1670* | Q9KRH2 | *OSU_1966* | L1QWP5 | 99 |
| Glucose-6-phosphate 1-dehydrogenase§ | *VC_A0896* | Q9KL52 | *OSU_2625* | L1QUN2 | 99 |
| Methylenetetrahydrofolate reductase§ | *VC_2685* | Q9KNP6 | *OSU_1236* | L1QZX5 | 100 |
| Arginine/ornithine succinyltransferase, putative§ | *VC_2617* | Q9KNW3 | *OSU_1168* | L1R056 | 99 |
| 6-carboxy-5,6,7,8-tetrahydropterin synthase§ | *VC_1299* | Q9KSF7 | *OSU_1022* | L1R0M7 | 100 |
| Phosphatidate cytidylyltransferase§ | *VC_1936* | Q9KQR2 | *OSU_3085* | L1QU85 | 99 |
| Phosphatidate cytidylyltransferase§ | *VC_2255* | Q9KPV7 | *OSU_3160* | L1QTH1 | 99 |
| Malate synthase§ | *VC_0734* | Q9KTZ6 | *OSU_0215* | L1R214 | 99 |
| Fructose-1,6-bisphosphatase§ | *VC_2688* | Q9KNP3 | *OSU_1238* | L1R0D6 | 100 |
| Ribulose-phosphate 3-epimerase§ | *VC_2625* | Q9KNV5 | *OSU_1178* | L1R066 | 99 |
| Amidophosphoribosyltransferase§ | *VC_1004* | Q9KT99 | *OSU_2719* | L1QVR0 | 99 |
| 50S ribosomal protein L31 type B¶ | *rpmE2* | Q9KTM4 | *rpmE2* | L1R2L2 | 100 |
| Peptidyl-tRNA hydrolase‖ | *pth* | Q9KQ21 | *pth* | L1QTF8 | 99 |
| Nucleoid occlusion factor SlmA‖ | *slmA* | Q9KVD2 | *slmA* | L1QSY9 | 100 |
| Probable septum site-determining protein MinC‖ | *minC* | Q9KQN9 | *minC* | L1QTF6 | 99 |
| RlpA-like lipoprotein‖ | *VC_0948* | Q9KTF4 | *OSU_0243* | L1R1G4 | 100 |
| DedD protein‖ | *VC_1002* | Q9KTA1 | *OSU_2717* | L1QV96 | 99 |
| DamX-related protein‖ | *VC_A0573* | Q9KM16 | *OSU_0886* | L1QZP0 | 100 |

| | | | | | |
|---|---|---|---|---|---|
| Cell division protein FtsN, putative‖ | *VC_2676* | Q9KNQ5 | *OSU_1226* | L1QZW5 | 99 |
| Site-specific recombinase IntI4‡ | *VC_A0291* | H9L4R7 | *OSU_0531* | L1R134 | 100 |

Listed genes are predicted to code for virulence, disease and defense, membrane transport, phages, prophages, transposable elements, plasmids, DNA metabolism, dormancy, sporulation, and regulons. In the table, the first column includes genes with their descriptions as per UniProt. Second and fourth columns represent abbreviated gene identifications for N16961 and PS15, respectively; the third and fifth columns represent accession numbers for N16961 and PS15, respectively. The % identity (fifth column) obtained from the BLAST analysis is the extent to which two sequences have the same residues at the same positions in an alignment. In the first column, candidates with (*) include proteins with putative functions in virulence, disease and defense. The symbols (†) and (§) include proteins that have functions in membrane transport and DNA metabolism, respectively; the symbol (‖) includes proteins which have functions within dormancy and sporulation categories, and (¶) includes proteins that are part of regulons. The symbol (‡) represents proteins that have functions within categories such as phages, prophages, transposable elements and plasmids.
